# Supplementary material for: Association of cardiometabolic risk factors with hospitalisation or death due to COVID-19: population-based cohort study in Sweden (SCAPIS)
Source: BMJ Open. 2021 Sep 2;11(9):e051359. doi: 10.1136/bmjopen-2021-051359 (PMC8413466; doi:10.1136/bmjopen-2021-051359)
Supplement: Supplementary data [file bmjopen-2021-051359supp001.pdf]

Supplementary Table 1 Definitions and categorization of selected variables.

Variable	Categorization	n (%) missing[a]	n (%) missing in main analyses[b]
**Sociodemographic information**			
Age	Continuous in years	0 (0)	936 (3.1)
Sex	1. Women<br>2. Men	0 (0)	936 (3.1)
Place of birth	1. Not born in Sweden<br>2. Born in Sweden	811 (2.7)	936 (3.1)
Education	1. High school or less<br>2. Vocational education<br>3. University	845 (2.8)	
**Cardiometabolic risk factors**			
Diabetes status	1. Normoglycemia<br>2. Prediabetes (fasting glucose [6.1-6.9 mmol/L or glycated hemoglobin ≥42 mmol/mol and <48 mmol/mol])<br>3. Diabetes diagnosis by physician (self-reported in questionnaire) or glycated hemoglobin ≥48 mmol/mol.	145 (0.5)	1064 (3.6)
Glycated hemoglobin	Continuous in mmol/mol	152 (0.5)	1070 (3.6)
Body mass index	Continuous in kg/m^2	2 (<0.5)	936 (3.1)
Weight status	1. Normal weight<br>2. Overweight<br>3. Obesity	2 (0)	936 (3.1)
Waist-hip ratio	Continuous	1567 (5.2)	2441 (8.1)
Systolic blood pressure	Continuous in mmHg	151 (0.5)	1022 (3.4)
Diastolic blood pressure	Continuous in mmHg	153 (0.5)	1024 (3.4)
Blood pressure level	Level as measured at inclusion in SCAPIS.<br>1. Normotensive (systolic blood pressure <140 mmHg and diastolic blood pressure <90 mmHg)<br>2. Grade 1 hypertension (Systolic blood pressure ≥140 mmHg and <160 mmHg or diastolic blood pressure ≥90 mmHg and <100 mmHg)<br>3. Grade 2 hypertension (systolic blood pressure ≥160 mmHg or diastolic blood pressure ≥100 mmHg).	153 (0.5)	1024 (3.4)
Current smoking	Self-reported in questionnaire.<br>1. No<br>2. Yes	954 (3.2)	1288 (4.3)
Time spent sedentary per day	Self-reported in questionnaire.<br>Continuous (hours per day)	10441 (34.9)	10475 (35)
Coronary artery calcium score by computer tomography	Continuous	1197 (4.0)	1992 (6.6)
Total cholesterol	Continuous in mmol/L	82 (0.3)	1004 (3.4)

Low-density lipoprotein (LDL) cholesterol	Continuous in mmol/L	218 (0.7)	1134 (3.8)
HDL cholesterol	Continuous in mmol/L	85 (0.3)	1005 (3.4)
Creatinine	Continuous in mikromol/L	61 (0.2)	984 (3.3)

a. n (%) missing values out of the total study population (n=29,955)
b. n (%) missing in the main analyses adjusted for age, sex, place of birth and education.

Supplementary Table 2 Characteristics of SCAPIS participants by their status of laboratory-confirmed diagnosis of Covid-19 between January 31 and September 12, 2020. Numbers are shown in n (%) unless otherwise indicated.

	Total study population	Laboratory-confirmed diagnosis of Covid-19
n		
	29955	299
Age, mean (SD)	61.2 (4.5)	60.3 (4.2)
Men	14535 (48.5)	127 (42.5)
Born outside of Sweden	4747 (16.3)	71 (24.1)
Education		
High School or less	2712 (9.3)	26 (8.9)
Vocational	13249 (45.5)	129 (44.0)
University	13149 (45.2)	138 (47.1)
Weight status		
Normal weight	10746 (35.9)	103 (34.4)
Overweight	12850 (42.9)	127 (42.5)
Obesity	6357 (21.2)	69 (23.1)
Body mass index in kg/m^2, mean (SD)	27.0 (4.5)	27.3 (4.7)
Diabetes status		
Normoglycemia	22806 (76.5)	224 (74.9)
Prediabetes	4747 (15.9)	46 (15.4)
Diabetes	2257 (7.6)	29 (9.7)
Waist-hip ratio, mean (SD)	0.9 (0.1)	0.9 (0.1)
Current smoking	3791 (13.1)	23 (8.0)
Sedentary time per day in hours, mean (SD)	6.9 (3.6)	6.6 (3.8)
Blood pressure level		
Normotensive	23335 (78.3)	235 (79.1)
Grade 1 hypertension	5141 (17.3)	48 (16.2)
Grade 2 hypertension	1326 (4.4)	14 (4.7)
Systolic blood pressure, mean (SD)	125.9 (17.0)	126.4 (17.9)
Diastolic blood pressure, mean (SD)	77.5 (10.5)	77.6 (10.7)
Triglycerides in mmol/L, mean (SD)	1.2 (0.8)	1.3 (0.9)
HDL in mmol/L, mean (SD)	1.6 (0.5)	1.6 (0.5)
Total cholesterol in mmol/L, mean (SD)	5.5 (1.1)	5.6 (1)
LDL cholesterol in mmol/L, mean (SD)	3.4 (1.0)	3.5 (0.9)
Glycated hemoglobin in mmol/mol, mean (SD)	36.6 (6.5)	36.8 (6.8)
Creatinine in µmol/L, mean (SD)	77.7 (16.5)	77.3 (22.6)
Coronary artery calcium score, mean (SD)	61.6 (229.5)	39.5 (144.4)
