# Supplementary material for: Association of cardiometabolic risk factors with hospitalisation or death due to COVID-19: population-based cohort study in Sweden (SCAPIS)
Source: BMJ Open. 2021 Sep 2;11(9):e051359. doi: 10.1136/bmjopen-2021-051359 (PMC8413466; doi:10.1136/bmjopen-2021-051359)
Supplement: Author's manuscript [file bmjopen-2021-051359.draft_revisions.pdf]

BMJ Open

BMJ Open is committed to open peer review. As part of this commitment we make the peer review history of every article we publish publicly available.

When an article is published we post the peer reviewers' comments and the authors' responses online. We also post the versions of the paper that were used during peer review. These are the versions that the peer review comments apply to.

The versions of the paper that follow are the versions that were submitted during the peer review process. They are not the versions of record or the final published versions. They should not be cited or distributed as the published version of this manuscript.

BMJ Open is an open access journal and the full, final, typeset and author-corrected version of record of the manuscript is available on our site with no access controls, subscription charges or pay-per-view fees (http://bmjopen.bmj.com).

If you have any questions on BMJ Open's open peer review process please email info.bmjopen@bmj.com

BMJ Open

Cardiometabolic risk factors and hospitalization or death due to COVID-19: population-based cohort study in Sweden (SCAPIS)

Journal:	*BMJ Open*
Manuscript ID	bmjopen-2021-051359
Article Type:	Original research
Date Submitted by the Author:	18-Mar-2021
Complete List of Authors:	Tornhammar, Per; Karolinska University Hospital, Functional Area of Emergency Medicine<br>Jernberg, Tomas; Karolinska Institutet, Department of Clinical Sciences, Danderyd University Hospital<br>Bergström, Göran; Sahlgrenska University Hospital, Clinical Physiology; University of Gothenburg Sahlgrenska Academy, Department of Molecular and Clinical Medicine<br>Blomberg, Anders; Umeå University, Department of Public Health and Clinical Medicine, Section of Medicine<br>Engström, Gunnar; Lund University, Department of Clinical Science in Malmö<br>Engvall, Jan; Linköping University, CMIV, Centre of Medical Image Science and Visualization; Linköping University, Department of Clinical Physiology, and Department of Health, Medicine and Caring Sciences,<br>Gisslén, Magnus; University of Gothenburg Sahlgrenska Academy, Department of Infectious Diseases, Institute of Biomedicine; Sahlgrenska University Hospital, Department of Infectious Diseases, Region Västra Götaland<br>Lind, Lars; Uppsala University, Department of Medical Sciences, Clinical Epidemiology<br>Sundström, Johan; Uppsala University, Department of Medical Sciences, Clinical Epidemiology; University of New South Wales, The George Institute for Global Health<br>Söderberg, Stefan ; Umeå University, Department of Public Health and Clinical Medicine, Section of Medicine<br>Zaigham, Suneela; Lund University, Department of Clinical Science in Malmö<br>Östgren, Carl Johan; Linköping University, Department of Health, Medicine and Caring Sciences<br>Andersson, Daniel Peter; Karolinska University Hospital, Department of Medicine Huddinge H7, Karolinska Institutet<br>Ueda, Peter; Karolinska Institutet, Clinical Epidemiology Division, Department of Medicine, Solna
Keywords:	COVID-19, Epidemiology < INFECTIOUS DISEASES, Cardiac Epidemiology < CARDIOLOGY

SCHOLARONE™
Manuscripts

I, the Submitting Author has the right to grant and does grant on behalf of all authors of the Work (as defined in the below author licence), an exclusive licence and/or a non-exclusive licence for contributions from authors who are: i) UK Crown employees; ii) where BMJ has agreed a CC-BY licence shall apply, and/or iii) in accordance with the terms applicable for US Federal Government officers or employees acting as part of their official duties; on a worldwide, perpetual, irrevocable, royalty-free basis to BMJ Publishing Group Ltd ("BMJ") its licensees and where the relevant Journal is co-owned by BMJ to the co-owners of the Journal, to publish the Work in this journal and any other BMJ products and to exploit all rights, as set out in our licence.

The Submitting Author accepts and understands that any supply made under these terms is made by BMJ to the Submitting Author unless you are acting as an employee on behalf of your employer or a postgraduate student of an affiliated institution which is paying any applicable article publishing charge ("APC") for Open Access articles. Where the Submitting Author wishes to make the Work available on an Open Access basis (and intends to pay the relevant APC), the terms of reuse of such Open Access shall be governed by a Creative Commons licence – details of these licences and which Creative Commons licence will apply to this Work are set out in our licence referred to above.

Other than as permitted in any relevant BMJ Author's Self Archiving Policies, I confirm this Work has not been accepted for publication elsewhere, is not being considered for publication elsewhere and does not duplicate material already published. I confirm all authors consent to publication of this Work and authorise the granting of this licence.

For peer review only

Cardiometabolic risk factors and hospitalization or death due to COVID-19: population-based cohort study in Sweden (SCAPIS)

Per Tornhammar[1], Tomas Jernberg[2], Göran Bergström[3,4], Anders Blomberg[5], Gunnar Engström[6], Jan Engvall[7,8], Magnus Gisslen[9,10], Lars Lind[11], Johan Sundström[11,12], Stefan Söderberg[5], Suneela Zaigham[6], Carl Johan Östgren[13], Daniel Peter Andersson[14*], Peter Ueda[15*]

*Shared last authorship

[1] Functional Area of Emergency Medicine, Karolinska University Hospital, Huddinge, Stockholm, Sweden

[2] Department of Clinical Sciences, Danderyd Hospital, Karolinska Institutet, Stockholm, Sweden.

[3] Department of Molecular and Clinical Medicine, Sahlgrenska Academy, University of Gothenburg, Gothenburg, Sweden

[4] Clinical Physiology, Sahlgrenska University Hospital, Gothenburg, Sweden

[5] Department of Public Health and Clinical Medicine, Section of Medicine, Umeå University, Umeå, Sweden

[6] Department of Clinical Science in Malmö, Lund University, 20502 Malmö, Sweden.

[7] CMIV, Centre of Medical Image Science and Visualization, Linköping University, Linköping, Sweden

[8] Department of Clinical Physiology, and Department of Health, Medicine and Caring Sciences, Linköping University, Linköping, Sweden

[9] Department of Infectious Diseases, Institute of Biomedicine, Sahlgrenska Academy, University of Gothenburg, Gothenburg, Sweden.

[10] Department of Infectious Diseases, Region Västra Götaland, Sahlgrenska University Hospital, Gothenburg, Sweden

[11] Department of Medical Sciences, Clinical Epidemiology, Uppsala University, Uppsala, Sweden

[12] The George Institute for Global Health, University of New South Wales, Sydney, Australia

[13] Department of Health, Medicine and Caring Sciences, Linköping University

For peer review only

[14] Department of Medicine Huddinge H7, Karolinska Institutet, Karolinska University Hospital, Stockholm, Sweden

[15] Clinical Epidemiology Division, Department of Medicine, Solna, Karolinska Institutet, Stockholm, Sweden.

Word count: 1993

Word count abstract: 270

Tables: 2 Tables

Online supplement: 2 Supplementary Tables

Correspondence: Peter Ueda, MD, PhD. Clinical Epidemiology Division, Department of Medicine, Solna, Eugeniahemmet, T2, Karolinska University Hospital, 17176, Stockholm, Sweden. Email: peter.ueda@ki.se

For peer review only

Abstract

Objective

To assess the association of cardiometabolic risk factors with hospitalization or death due to Covid-19 in the general population.

Design, setting and participants

Swedish population-based cohort including 29,955 participants.

Exposures

Cardiometabolic risk factors assessed between 2014 and 2018.

Main outcome measures

Hospitalization or death due to Covid-19, as registered in nationwide registers from January 31 through September 12, 2020. Associations of cardiometabolic risk factors with the outcome were assessed using logistic regression adjusted for age, sex, birthplace, and education.

Results

Mean [SD] age was 61.2 [4.5] and 51.5% were women. 69 participants experienced hospitalization or death due to Covid-19. Examples of statistically significant associations between baseline factors and subsequent hospitalization or death due to Covid-19 included overweight (adjusted odds ratio [aOR] vs normal weight 2.73 [95% CI 1.25-5.94]), obesity (aOR vs normal weight 4.09 [95% CI 1.82-9.18]), prediabetes (aOR vs normoglycemia 2.56 [95% CI 1.44-4.55]), diabetes (aOR vs normoglycemia 3.96 [95% CI 2.13-7.36]), sedentary time (aOR per hour/day increase 1.10 [95% CI 1.02-1.17]), grade 2 hypertension (aOR vs normotension 2.44 [95% CI 1.10-5.44]) and high density lipoprotein cholesterol (aOR per mmol/L increase 0.33 [95% CI 0.17-0.65]).

For peer review only

Statistically significant associations were not observed for grade 1 hypertension (aOR vs normotension 1.03 [95% CI 0.55-1.96]), current smoking (aOR 0.56 [95% CI 0.24-1.30]), total cholesterol (aOR per mmol/L increase 0.90 [95% CI 0.71-1.13]), low density lipoprotein cholesterol (aOR per mmol/L increase 0.90 [95% CI 0.69-1.15]) and coronary artery calcium score (aOR per 10 units increase 1.00 [95% CI 0.99-1.01]).

Conclusions

In a large population-based sample from the general population, several cardiometabolic risk factors were associated with hospitalization or death due to Covid-19.

For peer review only

Strengths and limitations of this study

- This study used data on cardiometabolic risk factors measured between 2014 and 2018 in a population-based cohort of over 30000 participants and assessed their association with hospitalization or death due to Covid-19 during the first wave of the pandemic.
- Few previous studies have used population-based samples from the general population to assess the relationship between cardiometabolic risk factors and outcomes in Covid-19.
- As we could not capture all cases of Covid-19 which did not lead to hospitalization or death, we could not assess to what extent the observed associations may reflect the relationship with exposure to SARS-CoV 2 as compared with the risk of hospitalization or death due to Covid-19 among those who have been exposed to the virus.

For peer review only

Identification of individuals at risk of worse outcomes in Covid-19 may inform risk management decisions to mitigate exposure and to prioritize vaccination. Several studies have shown an that cardiometabolic risk factors are associated with a higher risk of adverse outcomes in Covid-19. While many of these studies have been performed in selected populations such as patients hospitalized with Covid-19[1,2] or patients with certain diagnoses,[3,4] fewer studies have been based on data from the general population.[5–8]

The Swedish Cardiopulmonary bioimage study (SCAPIS)[9] is a population-based cohort of approximately 30,000 men and women who were extensively characterized with respect to cardiometabolic risk factors and function at the age of 50-64 years during the years preceding the Covid-19 outbreak (2014 through 2018). We used data from SCAPIS to assess the association of cardiometabolic risk factors with risk of hospitalization or death due to Covid-19 during the first wave of the pandemic.

METHODS

Data sources

The SCAPIS cohort, described in detail elsewhere,[9] is a population-based cohort conducted at six Swedish university hospitals in Gothenburg, Linköping, Malmö/Lund, Stockholm, Umeå and Uppsala, with each site recruiting participants from corresponding municipality areas. From 2014 through 2018, over 30,000 participants aged 50-64 years underwent examinations of cardiopulmonary risk factors and provided information regarding lifestyle factors and socioeconomic conditions. Using

For peer review only

the personal identity number, we linked SCAPIS data to nationwide administrative and health registers. From the National Patient Register, which comprises physician-assigned diagnoses based on the International Classification of Diseases, 10th Revision, Swedish Edition (ICD-10-SE) for all hospital admissions in Sweden, we obtained information about hospitalization for Covid-19. From the Cause of Death register, we obtained data about vital status, and date and cause of death. From SmiNet,[10] a reporting system for infectious diseases administered by the Public Health Agency, we obtained information about laboratory-confirmed cases of Covid-19, including those not leading to hospitalization or death.

Outcomes, exposures, and study period

The main outcome was hospitalization or death due to Covid-19. Hospitalization for Covid-19 was defined as hospital admission with laboratory-confirmed Covid-19 as the primary diagnosis (ICD-10-SE code U07.1 [Covid-19, virus identified]).[11] Death due to Covid-19 was defined as death where U07.1 was specified as the underlying cause of death. Follow-up was from January 31, 2020 (the first laboratory-confirmed case of Covid-19 in Sweden) through September 12, 2020.

We included variables in the SCAPIS dataset, (described in detail in Supplementary Table 1), that we hypothesized could be associated with hospitalization or death due to Covid-19: these included sociodemographic variables (age, sex, place of birth and education); diabetes status (normoglycemia, prediabetes, diabetes); weight status (normal [body mass index [BMI] <25 kg/m^2], overweight [BMI ≥25 to <30 kg/m^2], obesity [BMI ≥30 kg/m^2]); current smoking, waist-hip ratio, blood pressure status (normotension, grade 1 hypertension, grade 2 hypertension); systolic and diastolic

For peer review only

blood pressure; coronary artery calcium score; total cholesterol; low density lipoprotein (LDL) cholesterol; high density lipoprotein (HDL) cholesterol; glycated hemoglobin; and creatinine.

Study population

We included all 30,154 participants in SCAPIS. We excluded 199 participants who had missing data on vital status or died before January 31, 2020. The study population included 29,955 participants.

Statistical analysis

Analyses were performed in Stata 16.0. We described study participants with respect to the selected variables, separately among those who did not experience hospitalization or death due to Covid-19 and among those who did. We used logistic regression to assess the association between the investigated variables and hospitalization or death due to Covid-19, adjusting for sociodemographic variables including age (continuous variable), sex, place of birth (outside of Sweden vs Sweden) and education (high school or less, vocational, university).[12] Analyses were performed among those with complete data for each analyzed variable.

The relationship between the investigated variables and hospitalization or death due to Covid-19 may not only represent the risk of worse Covid-19 outcomes in individuals exposed to SARS-CoV-2 but also the risk of exposure to SARS-CoV-2. We therefore performed an additional analysis restricted to the subgroup of participants (n=299) with a laboratory-confirmed Covid-19 diagnosis as recorded in SmiNet, the National Patient Register or the Cause of Death Register (i.e., participants with a laboratory-

For peer review only

confirmed of Covid-19, including both those who did and did not experience hospitalization or death due to Covid-19). In this subgroup, we used logistic regression to assess the association of the selected variables and hospitalization or death due to Covid-19. Odds ratios whose 95% CI did not overlap 1 were considered as statistically significant. The Swedish Ethical Review Authority approved the study.

Patient involvement

No patients were involved in setting the research question, nor in the design, conduct, or interpretation of the study. The study findings are planned to be disseminated through the SCAPIS website.

Results

Characteristics of the 29,955 study participants are shown in Table 1. 14,535 (48.5%) were men. Mean (SD) age was 61.2 (4.5) years. 69 (0.2%) of the participants were hospitalized or died due to Covid-19. Those who experienced (n=69) vs did not experience (n=29,866) hospitalization or death due to Covid-19 were slightly older (61.9 years vs 61.2 years) and more likely to be men (75.4% vs 48.5%), born outside of Sweden (32.4% vs 16.3%) and to have an education of high school or less (17.9% vs 9.3%).

For peer review only

Table 1 Characteristics of SCAPIS participants by experience of hospitalization or death due to Covid-19 during between January 31 and September 12, 2020. Numbers are shown in n (%) unless otherwise indicated.

	Hospitalization or death due to Covid-19	
	No (n=29,866)	Yes (n=69)
Age, mean (SD)	61.2 (4.5)	61.9 (4.8)
Men	14483 (48.5)	52 (75.4)
Born outside of Sweden	4725 (16.3)	22 (32.4)
Education		
High School or less	2700 (9.3)	12 (17.9)
Vocational	13218 (45.5)	31 (46.3)
University	13125 (45.2)	24 (35.8)
Weight status		
Normal weight	10738 (35.9)	8 (11.6)
Overweight	12814 (42.9)	36 (52.2)
Obesity	6332 (21.2)	25 (36.2)
Body mass index in kg/m^2, mean (SD)	27.0 (4.5)	30.0 (5.3)
Diabetes status		
Normoglycemia	22774 (76.6)	32 (46.4)
Prediabetes	4727 (15.9)	20 (29.0)
Diabetes	2240 (7.5)	17 (24.6)
Waist-hip ratio, mean (SD)	0.92 (0.09)	0.98 (0.09)
Sedentary time per day in hours, mean (SD)	6.9 (3.5)	8.3 (4.5)
Blood pressure measurement		
Normotensive	23285 (78.3)	50 (72.5)
Grade 1 hypertension	5129 (17.3)	12 (17.4)
Grade 2 hypertension	1319 (4.4)	7 (10.1)
Systolic blood pressure, mean (SD)	125.9 (17.0)	133.5 (19.5)
Diastolic blood pressure, mean (SD)	77.5 (10.5)	81.0 (12.4)
Triglycerides in mmol/L, mean (SD)	1.2 (0.8)	1.6 (1.1)
HDL in mmol/L, mean (SD)	1.6 (0.5)	1.3 (0.4)
Total cholesterol in mmol/L, mean (SD)	5.5 (1.1)	5.3 (1.0)
LDL cholesterol in mmol/L, mean (SD)	3.4 (1.0)	3.3 (0.9)
Glycated hemoglobin in mmol/mol, mean (SD)	36.6 (6.4)	39.7 (9.3)
Creatinine in µmol/L, mean (SD)	77.7 (16.4)	86.2 (37.9)
Coronary artery calcium score, mean (SD)	61.6 (229.5)	90.2 (199.6)

For peer review only

The results of the logistic regression assessing the association between the selected variables and hospitalization or death due to Covid-19 (adjusted for age, sex, place of birth and education) are shown in Table 2. Significant associations were observed for overweight (adjusted odds ratio [aOR] vs normal weight 2.73 [95% CI 1.25 to 5.94]), obesity (aOR vs normal weight 4.09 [95% CI 1.82 to 9.18]), body mass index (aOR per 5 kg/m^2 increase 1.77 [95% CI 1.43 to 2.19]), prediabetes (aOR vs normoglycemia 2.56 [95% CI 1.44 to 4.55]), diabetes (aOR vs normoglycemia 3.96 [95% CI 2.13 to 7.36]), waist-hip ratio (aOR per SD increase 1.55 [95% CI 1.20 - 2.00]), sedentary time (aOR per hour/day increase 1.10 [95% CI 1.02 to 1.17]), grade 2 hypertension (aOR vs normotension 2.44 [95% CI 1.10 to 5.44]), systolic blood pressure (aOR per 10 mmHg increase 1.22 [95% CI 1.07 to 1.40]), diastolic blood pressure (aOR per 10 mmHg increase 1.31 [95% CI 1.05 to 1.64]), triglycerides (aOR per mmol/L increase 1.10 [95% CI 1.00 to 1.21]), HDL cholesterol (aOR per mmol/L increase 0.33 [95% CI 0.17 to 0.65]) and glycated hemoglobin (aOR per mmol/mol increase 1.03 [95% CI 1.01 to 1.05]). Significant associations were not observed for grade 1 hypertension (aOR vs normotension 1.03 [95% CI 0.55 to 1.96]), current smoking (aOR 0.56 [95% CI 0.24 to 1.30]), total cholesterol (aOR per mmol/L increase 0.90 [95% CI 0.71 to 1.13]), LDL cholesterol (aOR per mmol/L increase 0.90 [95% CI 0.69 to 1.15]), creatinine (aOR per 10 μmol/L increase 1.05 [95% CI 0.999 to 1.103]) and coronary artery calcium score (aOR per 10 units increase 1.00 [95% CI 0.99 to 1.01]).

For peer review only

Table 2 Odds ratios (95% CI)[a], adjusted for age, sex, place of birth and education, for the association of selected variables with hospitalization or death due to Covid-19 in the main and additional analyses.

	Main analysis (n=29,955)	Additional analysis restricted to those with laboratory-confirmed Covid-19 diagnosis (n=299)
Age per year	1.03 (0.97 - 1.08)	1.11 (1.04 - 1.20)
Men	3.39 (1.93 - 5.95)	6.62 (3.45 - 12.71)
Born outside of Sweden	2.40 (1.43 - 4.03)	1.59 (0.80 - 3.18)
Education [b]		
High School or less	1.00 (ref)	1.00 (ref)
Vocational	0.58 (0.30 - 1.14)	0.41 (0.16 - 1.09)
University	0.49 (0.24 - 0.99)	0.26 (0.10 - 0.70)
Weight status		
Normal weight	1.00 (ref)	1.00 (ref)
Overweight	2.73 (1.25 - 5.94)	3.41 (1.41 - 8.27)
Obesity	4.09 (1.82 - 9.18)	4.86 (1.86 - 12.71)
Body mass index per 5 kg/m^2 increase	1.77 (1.43 - 2.19)	2.05 (1.44 - 2.92)
Diabetes status		
Normoglycemia	1.00 (ref)	1.00 (ref)
Prediabetes	2.56 (1.44 - 4.55)	3.72 (1.68 - 8.28)
Diabetes	3.96 (2.13 - 7.36)	5.12 (1.95 - 13.42)
Waist-hip ratio per SD increase	1.55 (1.20 - 2.00)	1.92 (1.20 - 3.06)
Sedentary time per day per hour increase	1.10 (1.02 - 1.17)	1.13 (1.02 - 1.26)
Blood pressure measurement		
Normotensive	1.00 (ref)	1.00 (ref)
Grade 1 hypertension	1.03 (0.55 - 1.96)	0.95 (0.41 - 2.17)
Grade 2 hypertension	2.44 (1.10 - 5.44)	4.18 (1.07 - 16.32)
Systolic blood pressure per 10 mmHg increase	1.22 (1.07 - 1.40)	1.21 (1.01 - 1.44)
Diastolic blood pressure per 10 mmHg increase	1.31 (1.05 - 1.64)	1.30 (0.97 - 1.73)
Current smoking	0.56 (0.24 - 1.30)	0.87 (0.28 - 2.71)
Triglycerides per mmol/L increase	1.10 (1.00 - 1.21)	1.34 (0.98 - 1.85)
HDL per mmol/L increase	0.33 (0.17 - 0.65)	0.26 (0.10 - 0.66)
Total cholesterol per mmol/L increase	0.90 (0.71 - 1.13)	0.80 (0.59 - 1.10)
LDL cholesterol per mmol/L increase	0.90 (0.69 - 1.15)	0.83 (0.59 - 1.17)
Glycated hemoglobin per mmol/mol increase	1.03 (1.01 - 1.05)	1.04 (1.00 - 1.09)
Creatinine per 10 μmol/L increase	1.05 (1.00 - 1.10)	1.04 (0.88 - 1.23)
Coronary artery calcium score per 10 units increase	1.00 (0.99 - 1.01)	1.00 (0.98 - 1.02)

[a] Reference group for binary variables (Yes/No) is "No".
[b] Adjusted for sex and place of birth.

For peer review only

Population characteristics for the additional analysis including the 299 participants with laboratory confirmed Covid-19 are shown in Supplementary Table 2. The results of the logistic regression analyses are shown in Table 2. The findings were largely similar to those of the main analysis.

Discussion

We assessed the association of cardiometabolic risk factors with risk of hospitalization or death due to Covid-19 during the first wave of the pandemic in a Swedish population-based cohort with nearly 30,000 participants aged 52-72 years in 2020.

In analyses adjusted for age, sex, place of birth and education, we found that several cardiometabolic risk factors were associated with an increased risk of hospitalization or death due to Covid-19. Significant associations were found for metabolic risk factors, including overweight, obesity, prediabetes, diabetes and higher waist-hip ratio and glycated hemoglobin; findings that are in line with previous studies on these or related risk factors.[7,8,13] While significant associations were observed for grade 2 hypertension and for systolic and diastolic blood pressure when analyzed as continuous variables, grade 1 hypertension was not associated with an increased risk. Moreover, while lower HDL cholesterol and higher triglycerides were associated with hospitalization or death due to Covid-19, such associations were not observed for LDL cholesterol or total cholesterol. Associations with hospitalization or death due to Covid-19 were also not observed for current smoking and a higher coronary artery calcium score. Previous studies on the association of smoking, hypertension, lipid levels and

For peer review only - http://bmjopen.bmj.com/site/about/guidelines.xhtml

coronary artery calcium score with Covid-19 outcomes have yielded mixed findings.[8,14–18]

Strengths of our study include the use of a large sample from the general population who had undergone assessment of cardiometabolic risk factors in the years preceding the pandemic. Our study has limitations. Although the SCAPIS cohort includes detailed data on cardiometabolic risk factors from a large number of participants, the limited number of Covid-19 cases leading to hospitalization or death resulted in wide confidence intervals for some of the analyses. Moreover, during the first months of the pandemic, laboratory testing for Covid-19 was not widely performed and predominantly focused on healthcare professionals and hospitalized patients.[19] As such, although the findings of our analyses restricted to those with a laboratory-confirmed Covid-19 diagnosis were similar to those of our main analyses, we could not assess to what extent the observed associations may reflect the relationship with exposure to SARS-CoV 2 as compared with the risk of hospitalization or death due to Covid-19 among those who have been exposed to the virus. Updated analyses may be performed as data on broader testing for Covid-19 and more cases of hospitalization or death due to Covid-19 become available.

Conclusion

In this study large population-based cohort from the general population, several cardiometabolic risk factors were associated with hospitalization or death due to Covid-19.

For peer review only - http://bmjopen.bmj.com/site/about/guidelines.xhtml

ACKNOWLEDGEMENTS

Author Contributions

PU had full access to all the data in the study and take responsibility for the integrity of the data and the accuracy of the data analysis. PT, TJ, DA, and PU developed the research design. All authors contributed to the acquisition, analysis, or interpretation of data. DA and PU drafted first draft of the manuscript. All authors critically reviewed and revised the manuscript for important intellectual content and approved the manuscript. PU performed the statistical analysis. TJ and PU obtained funding.

Funding/Support

This work was supported by [Swedish Heart-Lung Foundation] grant number [20200491]. DPA was supported by [CIMED] grant number [20180855]. The main funding body of The Swedish CArdioPulmonary bioImage Study (SCAPIS) is the Swedish Heart-Lung Foundation. The study is also funded by the Knut and Alice Wallenberg Foundation, the Swedish Research Council and VINNOVA (Sweden's Innovation agency) the University of Gothenburg and Sahlgrenska University Hospital, Karolinska Institutet and Stockholm county council, Linköping University and University Hospital, Lund University and Skåne University Hospital, Umeå University and University Hospital, Uppsala University and University Hospital. Grant numbers are not applicable for the funding body of SCAPIS.

Role of the Funder/Sponsor

For peer review only

The funding sources had no role in the design and conduct of the study; collection, management, analysis, and interpretation of the data; preparation, review, or approval of the manuscript; and decision to submit the manuscript for publication.

Ethics approval

The study, complied with the Declaration of Helsinki, was approved by the Regional Ethical Review Board at Umeå University [2010-228-31M] and [2020-02668].

Conflict of Interest Disclosures

The authors have no conflict of interest to report.

Data sharing

No additional data available.

REFERENCES

1 Wu C, Chen X, Cai Y, *et al.* Risk Factors Associated With Acute Respiratory Distress Syndrome and Death in Patients With Coronavirus Disease 2019 Pneumonia in Wuhan, China. *JAMA Intern Med* Published Online First: 13 March 2020. doi:10.1001/jamainternmed.2020.0994

2 Docherty AB, Harrison EM, Green CA, *et al.* Features of 20 133 UK patients in hospital with covid-19 using the ISARIC WHO Clinical Characterisation Protocol: prospective observational cohort study. *BMJ* 2020;**369**:m1985. doi:10.1136/bmj.m1985

3 McGurnaghan SJ, Weir A, Bishop J, *et al.* Risks of and risk factors for COVID-19 disease in people with diabetes: a cohort study of the total population of Scotland. *Lancet Diabetes Endocrinol* Published Online First: 23 December 2020. doi:10.1016/S2213-8587(20)30405-8

4 Barron E, Bakhai C, Kar P, *et al.* Associations of type 1 and type 2 diabetes with

For peer review only

COVID-19-related mortality in England: a whole-population study. *Lancet Diabetes Endocrinol* 2020;**8**:813–22. doi:10.1016/S2213-8587(20)30272-2

5 Reilev M, Kristensen KB, Pottegård A, *et al.* Characteristics and predictors of hospitalization and death in the first 11 122 cases with a positive RT-PCR test for SARS-CoV-2 in Denmark: a nationwide cohort. *Int J Epidemiol* 2020;**49**:1468–81. doi:10.1093/ije/dyaa140

6 Petrilli CM, Jones SA, Yang J, *et al.* Factors associated with hospital admission and critical illness among 5279 people with coronavirus disease 2019 in New York City: prospective cohort study. *BMJ* 2020;**369**:m1966. doi:10.1136/bmj.m1966

7 Clift AK, Coupland CAC, Keogh RH, *et al.* Living risk prediction algorithm (QCOVID) for risk of hospital admission and mortality from coronavirus 19 in adults: national derivation and validation cohort study. *BMJ* 2020;**371**:m3731. doi:10.1136/bmj.m3731

8 Hamer M, Gale CR, Kivimäki M, *et al.* Overweight, obesity, and risk of hospitalization for COVID-19: A community-based cohort study of adults in the United Kingdom. *Proc Natl Acad Sci U S A* 2020;**117**:21011–3. doi:10.1073/pnas.2011086117

9 Bergström G, Berglund G, Blomberg A, *et al.* The Swedish CArdioPulmonary BioImage Study: objectives and design. *J Intern Med* 2015;**278**:645–59. doi:10.1111/joim.12384

10 Rolfhamre P, Janson A, Arneborn M, *et al.* SmiNet-2: Description of an internet-based surveillance system for communicable diseases in Sweden. *Eurosurveillance* 2006;**11**:15–6. doi:10.2807/esm.11.05.00626-en

11 Ludvigsson JF. The first eight months of Sweden's COVID-19 strategy and the key actions and actors that were involved. *Acta Paediatr Int J Paediatr* 2020;**109**:2459–71. doi:10.1111/apa.15582

12 Drefahl S, Wallace M, Mussino E, *et al.* A population-based cohort study of socio-demographic risk factors for COVID-19 deaths in Sweden. *Nat Commun* 2020;**11**:5097. doi:10.1038/s41467-020-18926-3

13 Xie J, Zu Y, Alkhatib A, *et al.* Metabolic Syndrome and COVID-19 Mortality Among Adult Black Patients in New Orleans. *Diabetes Care* 2021;**44**:188–93. doi:10.2337/dc20-1714

14 Garg H, Khanna P. Covid and cholesterol (C&C): Something to worry about or much ado about nothing? *Trends Anaesth Crit Care* Published Online First: September 2020. doi:10.1016/j.tacc.2020.09.003

15 Hopkinson NS, Rossi N, El-Sayed_Moustafa J, *et al.* Current smoking and COVID-19 risk: results from a population symptom app in over 2.4 million people. *Thorax* 2021;:thoraxjnl-2020-216422. doi:10.1136/thoraxjnl-2020-216422

16 Wenzl T. Smoking and COVID-19 - A review of studies suggesting a protective effect of smoking against COVID-19. EU Sci. Hub. doi:10.2760/564217

17 Dillinger JG, Benmessaoud FA, Pezel T, *et al.* Coronary Artery Calcification and

For peer review only - http://bmjopen.bmj.com/site/about/guidelines.xhtml

Complications in Patients With COVID-19. *JACC Cardiovasc Imaging* 2020;**13**:2468–70. doi:10.1016/j.jcmg.2020.07.004

18 Clark CE, McDonagh STJ, McManus RJ, *et al.* COVID-19 and hypertension: risks and management. A scientific statement on behalf of the British and Irish Hypertension Society. J. Hum. Hypertens. 2021;:1–4. doi:10.1038/s41371-020-00451-x

19 Ludvigsson JF. The first eight months of Sweden's COVID-19 strategy and the key actions and actors that were involved. *Acta Paediatr* 2020;:apa.15582. doi:10.1111/apa.15582

For peer review only

Supplementary Table 1 Definitions and categorization of selected variables.

Variable	Categorization
**Sociodemographic information**	
Age	Continuous in years
Sex	1. Women<br>2. Men
Place of birth	1. Not born in Sweden<br>2. Born in Sweden
Education	1. High school or less<br>2. Vocational education<br>3. University
**Cardiometabolic risk factors**	
Diabetes status	1. Normoglycemia<br>2. Prediabetes (fasting glucose [6.1-6.9 mmol/L or glycated hemoglobin ≥42 mmol/mol and <48 mmol/mol])<br>3. Diabetes diagnosis by physician (self-reported in questionnaire) or glycated hemoglobin ≥48 mmol/mol.
Glycated hemoglobin	Continuous in mmol/mol
Body mass index	Continuous in kg/m^2
Weight status	1. Normal weight<br>2. Overweight<br>3. Obesity
Waist-hip ratio	Continuous
Systolic blood pressure	Continuous in mmHg
Diastolic blood pressure	Continuous in mmHg
Blood pressure level	Level as measured at inclusion in SCAPIS.<br>1. Normotensive (systolic blood pressure <140 mmHg and diastolic blood pressure <90 mmHg)<br>2. Grade 1 hypertension (Systolic blood pressure ≥140 mmHg and <160 mmHg or diastolic blood pressure ≥90 mmHg and <100 mmHg)<br>3. Grade 2 hypertension (systolic blood pressure ≥160 mmHg or diastolic blood pressure ≥100 mmHg).
Current smoking	Self-reported in questionnaire.<br>1. No<br>2. Yes
Time spent sedentary per day	Self-reported in questionnaire.<br>Continuous (hours per day)
Coronary artery calcium score	Continuous
Total cholesterol	Continuous in mmol/L
Low-density lipoprotein (LDL) cholesterol	Continuous in mmol/L
HDL cholesterol	Continuous in mmol/L
Creatinine	Continuous in mikromol/L

For peer review only

Supplementary Table 2 Characteristics of SCAPIS participants with a laboratory-confirmed diagnosis (n=299) of Covid-19 between January 31 and September 12, 2020. Numbers are shown in n (%) unless otherwise indicated.

	Hospitalization or death due to Covid-19	
	No (n=230)	Yes (n=69)
Age, mean (SD)	59.9 (4)	61.9 (4.8)
Men	75 (32.6)	52 (75.4)
Born outside of Sweden	49 (21.7)	22 (32.4)
Education		
High School or less	14 (6.2)	12 (17.9)
Vocational	98 (43.4)	31 (46.3)
University	114 (50.4)	24 (35.8)
Weight status		
Normal weight	95 (41.3)	8 (11.6)
Overweight	91 (39.6)	36 (52.2)
Obesity	44 (19.1)	25 (36.2)
Body mass index in kg/m^2, mean (SD)	26.5 (4.2)	30 (5.3)
Diabetes status		
Normoglycemia	192 (83.5)	32 (46.4)
Prediabetes	26 (11.3)	20 (29)
Diabetes	12 (5.2)	17 (24.6)
Waist-hip ratio, mean (SD)	0.90 (0.08)	0.98 (0.09)
Sedentary time per day in hours, mean (SD)	6.1 (3.4)	8.3 (4.5)
Blood pressure level		
Normotensive	185 (81.1)	50 (72.5)
Grade 1 hypertension	36 (15.8)	12 (17.4)
Grade 2 hypertension	7 (3.1)	7 (10.1)
Systolic blood pressure, mean (SD)	124.2 (16.9)	133.5 (19.5)
Diastolic blood pressure, mean (SD)	76.6 (10.0)	81 (12.4)
Triglycerides in mmol/L, mean (SD)	1.2 (0.8)	1.6 (1.1)
HDL in mmol/L, mean (SD)	1.7 (0.5)	1.3 (0.4)
Total cholesterol in mmol/L, mean (SD)	5.6 (1.0)	5.3 (1.0)
LDL cholesterol in mmol/L, mean (SD)	3.6 (0.9)	3.3 (0.9)
Glycated hemoglobin in mmol/mol, mean (SD)	35.9 (5.6)	39.7 (9.3)
Creatinine in µmol/L, mean (SD)	74.6 (14.4)	86.2 (37.9)
Coronary artery calcium score, mean (SD)	24.4 (119.9)	90.2 (199.6)

For peer review only

STROBE Statement—Checklist of items that should be included in reports of ***cohort studies***

	Item No	Recommendation	Page
**Title and abstract**	1	(*a*) Indicate the study's design with a commonly used term in the title or the abstract	Title and abstract
		(*b*) Provide in the abstract an informative and balanced summary of what was done and what was found	Abstract
**Introduction**			
Background/rationale	2	Explain the scientific background and rationale for the investigation being reported	p.8
Objectives	3	State specific objectives, including any prespecified hypotheses	p.8
**Methods**			
Study design	4	Present key elements of study design early in the paper	p.8-10
Setting	5	Describe the setting, locations, and relevant dates, including periods of recruitment, exposure, follow-up, and data collection	p.8-10
Participants	6	(*a*) Give the eligibility criteria, and the sources and methods of selection of participants. Describe methods of follow-up	p. 10
		(*b*) For matched studies, give matching criteria and number of exposed and unexposed	
Variables	7	Clearly define all outcomes, exposures, predictors, potential confounders, and effect modifiers. Give diagnostic criteria, if applicable	p. 8-10 Supplementary Material (Supplementary Table 1).
Data sources/ measurement	8*	For each variable of interest, give sources of data and details of methods of assessment (measurement). Describe comparability of assessment methods if there is more than one group	8-10, Supplementary Table 1.
Bias	9	Describe any efforts to address potential sources of bias	p. 8-10
Study size	10	Explain how the study size was arrived at	p.10
Quantitative variables	11	Explain how quantitative variables were handled in the analyses. If applicable, describe which groupings were chosen and why	p.9-10, Supplementary Table 1
Statistical methods	12	(*a*) Describe all statistical methods, including those used to control for confounding	p.10-11
		(*b*) Describe any methods used to examine subgroups and interactions	p.10-11
		(*c*) Explain how missing data were addressed	p.10
		(*d*) If applicable, explain how loss to follow-up was addressed	NA
		(*e*) Describe any sensitivity analyses	NA
**Results**			
Participants	13*	(a) Report numbers of individuals at each stage of study—eg numbers potentially eligible, examined for eligibility, confirmed eligible, included in the study, completing follow-up, and analysed	p.10
		(b) Give reasons for non-participation at each stage	p.10
		(c) Consider use of a flow diagram	NA
Descriptive data	14*	(a) Give characteristics of study participants (eg demographic, clinical, social) and information on exposures and potential	Table 1

For peer review only - http://bmjopen.bmj.com/site/about/guidelines.xhtml

		confounders	
		(b) Indicate number of participants with missing data for each variable of interest	Table 1
		(c) Summarise follow-up time (eg, average and total amount)	p.10
Outcome data	15*	Report numbers of outcome events or summary measures over time	p.11
Main results	16	(*a*) Give unadjusted estimates and, if applicable, confounder-adjusted estimates and their precision (eg, 95% confidence interval). Make clear which confounders were adjusted for and why they were included	p.10-13, Table 1, Table 2
		(*b*) Report category boundaries when continuous variables were categorized	Supplementary Table 1
		(*c*) If relevant, consider translating estimates of relative risk into absolute risk for a meaningful time period	Not deemed relevant
Other analyses	17	Report other analyses done—eg analyses of subgroups and interactions, and sensitivity analyses	p. 10-13
**Discussion**			
Key results	18	Summarise key results with reference to study objectives	p.15
Limitations	19	Discuss limitations of the study, taking into account sources of potential bias or imprecision. Discuss both direction and magnitude of any potential bias	p.16
Interpretation	20	Give a cautious overall interpretation of results considering objectives, limitations, multiplicity of analyses, results from similar studies, and other relevant evidence	p.15
Generalisability	21	Discuss the generalisability (external validity) of the study results	p.15-16
**Other information**			
Funding	22	Give the source of funding and the role of the funders for the present study and, if applicable, for the original study on which the present article is based	p.17

*Give information separately for exposed and unexposed groups.

Note: An Explanation and Elaboration article discusses each checklist item and gives methodological background and published examples of transparent reporting. The STROBE checklist is best used in conjunction with this article (freely available on the Web sites of PLoS Medicine at http://www.plosmedicine.org/, Annals of Internal Medicine at http://www.annals.org/, and Epidemiology at http://www.epidem.com/). Information on the STROBE Initiative is available at http://www.strobe-statement.org.

For peer review only

BMJ Open

Association of cardiometabolic risk factors with hospitalization or death due to COVID-19: population-based cohort study in Sweden (SCAPIS)

Journal:	*BMJ Open*
Manuscript ID	bmjopen-2021-051359.R1
Article Type:	Original research
Date Submitted by the Author:	02-Jul-2021
Complete List of Authors:	Tornhammar, Per; Karolinska University Hospital, Functional Area of Emergency Medicine<br>Jernberg, Tomas; Karolinska Institutet, Department of Clinical Sciences, Danderyd University Hospital<br>Bergström, Göran; Sahlgrenska University Hospital, Clinical Physiology; University of Gothenburg Sahlgrenska Academy, Department of Molecular and Clinical Medicine<br>Blomberg, Anders; Umeå University, Department of Public Health and Clinical Medicine, Section of Medicine<br>Engström, Gunnar; Lund University, Department of Clinical Science in Malmö<br>Engvall, Jan; Linköping University, CMIV, Centre of Medical Image Science and Visualization; Linköping University, Department of Clinical Physiology, and Department of Health, Medicine and Caring Sciences,<br>Fall, Tove; Uppsala University, Medical Sciences<br>Gisslén, Magnus; University of Gothenburg Sahlgrenska Academy, Department of Infectious Diseases, Institute of Biomedicine; Sahlgrenska University Hospital, Department of Infectious Diseases, Region Västra Götaland<br>Janson, Christer; Uppsala University, Dep om Medical Sciences: Respiratory Medicine<br>Lind, Lars; Uppsala University, Department of Medical Sciences, Clinical Epidemiology<br>Skold, Magnus; Karolinska Institute, Respiratory Medicine; Karolinska University Hospital, Respiratory Medicine and Allergy<br>Sundström, Johan; Uppsala University, Department of Medical Sciences, Clinical Epidemiology; University of New South Wales, The George Institute for Global Health<br>Söderberg, Stefan ; Umeå University, Department of Public Health and Clinical Medicine, Section of Medicine<br>Zaigham, Suneela; Lund University, Department of Clinical Science in Malmö<br>Östgren, Carl Johan; Linköping University, Department of Health, Medicine and Caring Sciences<br>Andersson, Daniel Peter; Karolinska University Hospital, Department of Medicine Huddinge H7, Karolinska Institutet<br>Ueda, Peter; Karolinska Institutet, Clinical Epidemiology Division, Department of Medicine, Solna

**Primary Subject Heading**:	Epidemiology
Secondary Subject Heading:	Cardiovascular medicine, Public health
Keywords:	COVID-19, Epidemiology < INFECTIOUS DISEASES, Cardiac Epidemiology < CARDIOLOGY

SCHOLARONE™ Manuscripts

I, the Submitting Author has the right to grant and does grant on behalf of all authors of the Work (as defined in the below author licence), an exclusive licence and/or a non-exclusive licence for contributions from authors who are: i) UK Crown employees; ii) where BMJ has agreed a CC-BY licence shall apply, and/or iii) in accordance with the terms applicable for US Federal Government officers or employees acting as part of their official duties; on a worldwide, perpetual, irrevocable, royalty-free basis to BMJ Publishing Group Ltd ("BMJ") its licensees and where the relevant Journal is co-owned by BMJ to the co-owners of the Journal, to publish the Work in this journal and any other BMJ products and to exploit all rights, as set out in our licence.

The Submitting Author accepts and understands that any supply made under these terms is made by BMJ to the Submitting Author unless you are acting as an employee on behalf of your employer or a postgraduate student of an affiliated institution which is paying any applicable article publishing charge ("APC") for Open Access articles. Where the Submitting Author wishes to make the Work available on an Open Access basis (and intends to pay the relevant APC), the terms of reuse of such Open Access shall be governed by a Creative Commons licence – details of these licences and which Creative Commons licence will apply to this Work are set out in our licence referred to above.

Other than as permitted in any relevant BMJ Author's Self Archiving Policies, I confirm this Work has not been accepted for publication elsewhere, is not being considered for publication elsewhere and does not duplicate material already published. I confirm all authors consent to publication of this Work and authorise the granting of this licence.

For peer review only

Association of cardiometabolic risk factors with hospitalization or death due to COVID-19: population-based cohort study in Sweden (SCAPIS)

Per Tornhammar[1], Tomas Jernberg[2], Göran Bergström[3,4], Anders Blomberg[5], Gunnar Engström[6], Jan Engvall[7,8], Tove Fall[9], Magnus Gisslen[10,11], Christer Janson[12], Lars Lind[13], Magnus Sköld[14,15], Johan Sundström[13,16], Stefan Söderberg[5], Suneela Zaigham[6], Carl Johan Östgren[17], Daniel Peter Andersson[18]*, Peter Ueda[19]*

*Shared last authorship

[1] Functional Area of Emergency Medicine, Karolinska University Hospital, Huddinge, Stockholm, Sweden.

[2] Department of Clinical Sciences, Danderyd Hospital, Karolinska Institutet, Stockholm, Sweden.

[3] Department of Molecular and Clinical Medicine, Sahlgrenska Academy, University of Gothenburg, Gothenburg, Sweden.

[4] Clinical Physiology, Sahlgrenska University Hospital, Gothenburg, Sweden.

[5] Department of Public Health and Clinical Medicine, Section of Medicine, Umeå University, Umeå, Sweden.

[6] Department of Clinical Science in Malmö, Lund University, 20502 Malmö, Sweden.

[7] CMIV, Centre of Medical Image Science and Visualization, Linköping University, Linköping, Sweden.

[8] Department of Clinical Physiology, and Department of Health, Medicine and Caring Sciences, Linköping University, Linköping, Sweden.

[9] Department of Medical Sciences, Molecular Epidemiology and Science for Life Laboratory, Uppsala University, Uppsala, Sweden.

[10] Department of Infectious Diseases, Institute of Biomedicine, Sahlgrenska Academy, University of Gothenburg, Gothenburg, Sweden.

[11] Department of Infectious Diseases, Region Västra Götaland, Sahlgrenska University Hospital, Gothenburg, Sweden.

[12] Department of Medical Sciences Respiratory, Allergy and Sleep Research, Uppsala University, Uppsala, Sweden.

[13] Department of Medical Sciences, Clinical Epidemiology, Uppsala University, Uppsala, Sweden.

For peer review only

[14] Department of Respiratory Medicine and Allergy, Karolinska University Hospital Solna, Stockholm, Sweden.

[15] Respiratory Medicine Unit, Department of Medicine Solna and Center for Molecular Medicine, Karolinska Institutet, Stockholm, Sweden.

[16] The George Institute for Global Health, University of New South Wales, Sydney, Australia.

[17] Department of Health, Medicine and Caring Sciences, Linköping University, Linköping, Sweden.

[18] Department of Medicine Huddinge H7, Karolinska Institutet, Karolinska University Hospital, Stockholm, Sweden.

[19] Clinical Epidemiology Division, Department of Medicine, Solna, Karolinska Institutet, Stockholm, Sweden.

Word count: 1602

Word count abstract: 271

Tables: 2 Tables

Online supplement: 2 Supplementary Tables

Correspondence: Peter Ueda, MD, PhD. Clinical Epidemiology Division, Department of Medicine, Solna, Eugeniahemmet, T2, Karolinska University Hospital, 17176, Stockholm, Sweden. Email: peter.ueda@ki.se

For peer review only

Abstract

Objective

To assess the association of cardiometabolic risk factors with hospitalization or death due to Covid-19 in the general population.

Design, setting and participants

Swedish population-based cohort including 29,955 participants.

Exposures

Cardiometabolic risk factors assessed between 2014 and 2018.

Main outcome measures

Hospitalization or death due to Covid-19, as registered in nationwide registers from January 31 2020 through September 12, 2020. Associations of cardiometabolic risk factors with the outcome were assessed using logistic regression adjusted for age, sex, birthplace, and education.

Results

Mean [SD] age was 61.2 [4.5] and 51.5% were women. 69 participants experienced hospitalization or death due to Covid-19. Examples of statistically significant associations between baseline factors and subsequent hospitalization or death due to Covid-19 included overweight (adjusted odds ratio [aOR] vs normal weight 2.73 [95% CI 1.25-5.94]), obesity (aOR vs normal weight 4.09 [95% CI 1.82-9.18]), prediabetes (aOR vs normoglycemia 2.56 [95% CI 1.44-4.55]), diabetes (aOR vs normoglycemia 3.96 [95% CI 2.13-7.36]), sedentary time (aOR per hour/day increase 1.10 [95% CI 1.02-1.17]), grade 2 hypertension (aOR vs normotension 2.44 [95% CI 1.10-5.44]) and high density lipoprotein cholesterol (aOR per mmol/L increase 0.33 [95% CI 0.17-0.65]).

For peer review only

Statistically significant associations were not observed for grade 1 hypertension (aOR vs normotension 1.03 [95% CI 0.55-1.96]), current smoking (aOR 0.56 [95% CI 0.24-1.30]), total cholesterol (aOR per mmol/L increase 0.90 [95% CI 0.71-1.13]), low density lipoprotein cholesterol (aOR per mmol/L increase 0.90 [95% CI 0.69-1.15]) and coronary artery calcium score (aOR per 10 units increase 1.00 [95% CI 0.99-1.01]).

Conclusions

In a large population-based sample from the general population, several cardiometabolic risk factors were associated with hospitalization or death due to Covid-19.

For peer review only

Strengths and limitations of this study

- This study used data on cardiometabolic risk factors measured between 2014 and 2018 in a population-based cohort of over 30,000 participants and assessed their association with hospitalization or death due to Covid-19 during the first wave of the pandemic.
- Few previous studies have used population-based samples from the general population to assess the relationship between cardiometabolic risk factors and outcomes in Covid-19.
- As we could not capture all cases of Covid-19 which did not lead to hospitalization or death, we could not assess to what extent the observed associations may reflect the relationship with exposure to SARS-CoV 2 as compared with the risk of hospitalization or death due to Covid-19 among those who have been exposed to the virus.

For peer review only

Identification of individuals at risk of worse outcomes in Covid-19 may inform risk management decisions to mitigate exposure and to prioritize vaccination. Several studies have shown an that cardiometabolic risk factors are associated with a higher risk of adverse outcomes in Covid-19. While many of these studies have been performed in selected populations such as patients hospitalized with Covid-19[1,2] or patients with certain diagnoses,[3,4] fewer studies have been based on data from the general population.[5–8]

The Swedish Cardiopulmonary bioimage study (SCAPIS)[9] is a population-based cohort of approximately 30,000 men and women who were extensively characterized with respect to cardiometabolic risk factors and function at the age of 50-64 years during the years preceding the Covid-19 outbreak (2014 through 2018). We used data from SCAPIS to assess the association of cardiometabolic risk factors with risk of hospitalization or death due to Covid-19 during the first wave of the pandemic.

METHODS

Data sources

The SCAPIS cohort, described in detail elsewhere,[9] is a population-based cohort conducted at six Swedish university hospitals in Gothenburg, Linköping, Malmö/Lund, Stockholm, Umeå and Uppsala, with each site recruiting participants from corresponding municipality areas. From 2014 through 2018, over 30,000 participants aged 50-64 years underwent examinations of cardiopulmonary risk factors and provided information regarding lifestyle factors and socioeconomic conditions. Using

For peer review only

the personal identity number, we linked SCAPIS data to nationwide administrative and health registers. From the National Patient Register, which comprises physician-assigned diagnoses based on the International Classification of Diseases, 10th Revision, Swedish Edition (ICD-10-SE) for all hospital admissions in Sweden, we obtained information about hospitalization for Covid-19. From the Cause of Death register, we obtained data about vital status, and date and cause of death. From SmiNet,[10] a reporting system for infectious diseases administered by the Public Health Agency, we obtained information about laboratory-confirmed cases of Covid-19, including those not leading to hospitalization or death.

Outcomes, exposures, and study period

The main outcome was a composite of hospitalization due to Covid-19 and death due to Covid-19. Hospitalization for Covid-19 was defined as hospital admission with laboratory-confirmed Covid-19 as the primary diagnosis (ICD-10-SE code U07.1 [Covid-19, virus identified]).[11] Death due to Covid-19 was defined as death where U07.1 was specified as the underlying cause of death. Follow-up was from January 31, 2020 (the first laboratory-confirmed case of Covid-19 in Sweden) through September 12, 2020.

We included variables in the SCAPIS dataset, (described in detail in Supplementary Table 1), that we hypothesized could be associated with hospitalization or death due to Covid-19: these included sociodemographic variables (age, sex, place of birth and education) and cardiometabolic risk factors: diabetes status (normoglycemia, prediabetes, diabetes); weight status (normal [body mass index [BMI] <25 kg/m^2], overweight [BMI ≥25 to <30 kg/m^2], obesity [BMI ≥30 kg/m^2]); current smoking, waist-hip ratio, blood pressure status (normotension, grade 1 hypertension, grade 2

hypertension); systolic and diastolic blood pressure; coronary artery calcium score; total cholesterol; low density lipoprotein (LDL) cholesterol; high density lipoprotein (HDL) cholesterol; glycated hemoglobin; and creatinine. Few (≤2%) participants had established cardiovascular disease, including coronary heart disease, stroke and heart failure; therefore, we did not assess these variables.

Study population

We included all 30,154 participants in SCAPIS. We excluded 2 participants who had missing data on vital status and 197 participants who died before January 31, 2020. The study population included 29,955 participants.

Statistical analysis

Analyses were performed in Stata 16.0. We described study participants with respect to the selected variables, separately among those who did not experience hospitalization or death due to Covid-19 and among those who did and assessed differences between the groups using the Chi-squared test for categorical variables and the t-test for continuous variables. For each investigated variable separately, we used logistic regression to assess its association with hospitalization or death due to Covid-19, adjusting for sociodemographic variables including age (continuous variable), sex, place of birth (outside of Sweden vs Sweden) and education (high school or less, vocational, university).[12] Analyses were performed among those with complete data for all variables included in the model. The proportion of missing data for the analyzed variables are shown in Supplementary Table 1 and ranged between 0 and 5%; the exception was time spent sedentary per day for which 34.9% of the participants had missing data.

For peer review only

The relationship between the investigated variables and hospitalization or death due to Covid-19 may not only represent the risk of worse Covid-19 outcomes in individuals exposed to SARS-CoV-2 but also the risk of exposure to SARS-CoV-2. We therefore performed an additional analysis restricted to the subgroup of participants (n=299) with a laboratory-confirmed Covid-19 diagnosis as recorded in SmiNet, the National Patient Register or the Cause of Death Register (i.e., participants with a laboratory-confirmed of Covid-19, including both those who did and did not experience hospitalization or death due to Covid-19). In this subgroup, we used logistic regression to assess the association of each of the cardiometabolic risk factors (separately) with hospitalization or death due to Covid-19. Odds ratios whose 95% CI did not overlap 1 were considered as statistically significant. The Swedish Ethical Review Authority approved the study.

Patient involvement

No patients were involved in setting the research question, nor in the design, conduct, or interpretation of the study. The study findings are planned to be disseminated through the SCAPIS website.

RESULTS

Characteristics of the 29,955 study participants are shown in Table 1. 14,535 (48.5%) were men. Mean (SD) age was 61.2 (4.5) years. 69 (0.2%) of the participants were hospitalized or died due to Covid-19. Those who experienced (n=69) vs did not experience (n=29,866) hospitalization or death due to Covid-19 were slightly older

For peer review only

(61.9 years vs 61.2 years) and more likely to be men (75.4% vs 48.5%), born outside of Sweden (32.4% vs 16.3%) and to have education of high school or less (17.9% vs 9.3%).

Table 1 Characteristics of SCAPIS participants by experience of hospitalization or death due to Covid-19 between January 31 and September 12, 2020. Numbers are shown in n (%) unless otherwise indicated.

	Hospitalization or death due to Covid-19		
	No (n=29,866)	Yes (n=69)	P-value
Age, mean (SD)	61.2 (4.5)	61.9 (4.8)	0.226
Men	14483 (48.5)	52 (75.4)	<0.001
Born outside of Sweden	4725 (16.3)	22 (32.4)	<0.001
Education			
High School or less	2700 (9.3)	12 (17.9)	
Vocational	13218 (45.5)	31 (46.3)	
University	13125 (45.2)	24 (35.8)	0.036
Weight status			
Normal weight	10738 (35.9)	8 (11.6)	
Overweight	12814 (42.9)	36 (52.2)	
Obesity	6332 (21.2)	25 (36.2)	<0.001
Body mass index in kg/m^2, mean (SD)	27.0 (4.5)	30.0 (5.3)	<0.001
Diabetes status			
Normoglycemia	22774 (76.6)	32 (46.4)	
Prediabetes	4727 (15.9)	20 (29.0)	
Diabetes	2240 (7.5)	17 (24.6)	<0.001
Waist-hip ratio, mean (SD)	0.92 (0.09)	0.98 (0.09)	<0.001
Current smoking	3785 (13.1)	6 (9)	0.317
Sedentary time per day in hours, mean (SD)	6.9 (3.5)	8.3 (4.5)	0.009
Blood pressure measurement			
Normotensive	23285 (78.3)	50 (72.5)	
Grade 1 hypertension	5129 (17.3)	12 (17.4)	
Grade 2 hypertension	1319 (4.4)	7 (10.1)	0.069
Systolic blood pressure, mean (SD)	125.9 (17.0)	133.5 (19.5)	<0.001
Diastolic blood pressure, mean (SD)	77.5 (10.5)	81.0 (12.4)	0.006
Triglycerides in mmol/L, mean (SD)	1.2 (0.8)	1.6 (1.1)	<0.001
HDL in mmol/L, mean (SD)	1.6 (0.5)	1.3 (0.4)	<0.001
Total cholesterol in mmol/L, mean (SD)	5.5 (1.1)	5.3 (1.0)	0.068
LDL cholesterol in mmol/L, mean (SD)	3.4 (1.0)	3.3 (0.9)	0.360
Glycated hemoglobin in mmol/mol, mean (SD)	36.6 (6.4)	39.7 (9.3)	<0.001
Creatinine in µmol/L, mean (SD)	77.7 (16.4)	86.2 (37.9)	<0.001
Coronary artery calcium score, mean (SD)	61.6 (229.5)	90.2 (199.6)	0.053[a]

[a] Because 17016 of the participants had a score of 0, the p-value calculated using the Chi-squared with five categories: 0 and quartiles of score among participants with a score of >0.

For peer review only

The results of the logistic regression assessing the association between each of the selected cardiometabolic risk factors and hospitalization or death due to Covid-19 (adjusted for age, sex, place of birth and education) are shown in Table 2. Significant associations were observed for overweight and obesity, higher body mass index, prediabetes, diabetes, higher waist-hip ratio, more time spent sedentary per day, grade 2 hypertension, as well as higher systolic blood pressure, diastolic blood pressure, triglycerides and glycated hemoglobin, and lower HDL cholesterol. Significant associations were not observed for grade 1 hypertension, current smoking, total cholesterol, LDL cholesterol, creatinine and coronary artery calcium score.

For peer review only

Table 2 Odds ratios (95% CI)[a], adjusted for age, sex, place of birth and education, for the association of selected variables with hospitalization or death due to Covid-19 in the main and additional analyses.

	Main analysis (n=29,955)	Additional analysis restricted to those with laboratory-confirmed Covid-19 diagnosis (n=299)
Age per year	1.03 (0.97 - 1.08)	1.11 (1.04 - 1.20)
Men	3.39 (1.93 - 5.95)	6.62 (3.45 - 12.71)
Born outside of Sweden	2.40 (1.43 - 4.03)	1.59 (0.80 - 3.18)
Education [b]		
High School or less	1.00 (ref)	1.00 (ref)
Vocational	0.58 (0.30 - 1.14)	0.41 (0.16 - 1.09)
University	0.49 (0.24 - 0.99)	0.26 (0.10 - 0.70)
Weight status		
Normal weight	1.00 (ref)	1.00 (ref)
Overweight	2.73 (1.25 - 5.94)	3.41 (1.41 - 8.27)
Obesity	4.09 (1.82 - 9.18)	4.86 (1.86 - 12.71)
Body mass index per 5 kg/m^2 increase	1.77 (1.43 - 2.19)	2.05 (1.44 - 2.92)
Diabetes status		
Normoglycemia	1.00 (ref)	1.00 (ref)
Prediabetes	2.56 (1.44 - 4.55)	3.72 (1.68 - 8.28)
Diabetes	3.96 (2.13 - 7.36)	5.12 (1.95 - 13.42)
Waist-hip ratio per SD increase	1.55 (1.20 - 2.00)	1.92 (1.20 - 3.06)
Sedentary time per day per hour increase	1.10 (1.02 - 1.17)	1.13 (1.02 - 1.26)
Blood pressure measurement		
Normotensive	1.00 (ref)	1.00 (ref)
Grade 1 hypertension	1.03 (0.55 - 1.96)	0.95 (0.41 - 2.17)
Grade 2 hypertension	2.44 (1.10 - 5.44)	4.18 (1.07 - 16.32)
Systolic blood pressure per 10 mmHg increase	1.22 (1.07 - 1.40)	1.21 (1.01 - 1.44)
Diastolic blood pressure per 10 mmHg increase	1.31 (1.05 - 1.64)	1.30 (0.97 - 1.73)
Current smoking	0.56 (0.24 - 1.30)	0.87 (0.28 - 2.71)
Triglycerides per mmol/L increase	1.10 (1.00 - 1.21)	1.34 (0.98 - 1.85)
HDL per mmol/L increase	0.33 (0.17 - 0.65)	0.26 (0.10 - 0.66)
Total cholesterol per mmol/L increase	0.90 (0.71 - 1.13)	0.80 (0.59 - 1.10)
LDL cholesterol per mmol/L increase	0.90 (0.69 - 1.15)	0.83 (0.59 - 1.17)
Glycated hemoglobin per mmol/mol increase	1.03 (1.01 - 1.05)	1.04 (1.00 - 1.09)
Creatinine per 10 µmol/L increase	1.05 (1.00 - 1.10)	1.04 (0.88 - 1.23)
Coronary artery calcium score per 10 units increase	1.00 (0.99 - 1.01)	1.00 (0.98 - 1.02)

[a] Reference group for binary variables (Yes/No) is “No”.

[b] Adjusted for sex and place of birth.

For peer review only

Population characteristics for the additional analysis including the 299 participants with laboratory confirmed Covid-19 are shown in Supplementary Table 2. Compared to those without a laboratory-confirmed diagnosis of Covid-19, those with a laboratory-confirmed diagnosis but who did not experience hospitalization or death due to Covid-19 were less likely to be men, be current smokers and to have diabetes and more likely to be born outside of Sweden and to have a university education and a BMI within the normal range. The results of the logistic regression analyses are shown in Table 2. The findings were largely similar to those of the main analysis.

Discussion

We assessed the association of cardiometabolic risk factors with risk of hospitalization or death due to Covid-19 during the first wave of the pandemic in a Swedish population-based cohort with nearly 30,000 participants aged 52-72 years in 2020.

In analyses adjusted for age, sex, place of birth and education, we found that several cardiometabolic risk factors were associated with an increased risk of hospitalization or death due to Covid-19. Significant associations were found for metabolic risk factors, including overweight, obesity, prediabetes, diabetes and higher waist-hip ratio and glycated hemoglobin; findings that are in line with previous studies on these or related risk factors.[7,8,13] While significant associations were observed for grade 2 hypertension and for systolic and diastolic blood pressure when analyzed as continuous variables, grade 1 hypertension was not associated with an increased risk. Moreover, while lower HDL cholesterol and higher triglycerides were associated with hospitalization or death due to Covid-19, such associations were not observed for LDL

For peer review only

cholesterol or total cholesterol. Associations with hospitalization or death due to Covid-19 were also not observed for current smoking and a higher coronary artery calcium score. Previous studies on the association of smoking, hypertension, lipid levels and coronary artery calcium score with Covid-19 outcomes have yielded mixed findings.[8,14–18]

Strengths of our study include the use of a large sample from the general population who had undergone assessment of cardiometabolic risk factors in the years preceding the pandemic. Our study has limitations. Although the SCAPIS cohort includes detailed data on cardiometabolic risk factors from a large number of participants, the limited number of Covid-19 cases leading to hospitalization or death resulted in wide confidence intervals for some of the analyses. Moreover, during the first months of the pandemic, laboratory testing for Covid-19 was not widely performed and predominantly focused on healthcare professionals and hospitalized patients.[19] As such, although the findings of our analyses restricted to those with a laboratory-confirmed Covid-19 diagnosis were similar to those of our main analyses (although the analyses were based on a small sample of 299 participants), we could not assess to what extent the observed associations may reflect the relationship with exposure to SARS-CoV 2 as compared with the risk of hospitalization or death due to Covid-19 among those who have been exposed to the virus. Updated analyses may be performed as data on broader testing for Covid-19 and more cases of hospitalization or death due to Covid-19 become available. Finally, the study included individuals in a limited age range.

Conclusion

For peer review only - http://bmjopen.bmj.com/site/about/guidelines.xhtml

In this study large population-based cohort from the general population, several cardiometabolic risk factors were associated with hospitalization or death due to Covid-19.

For peer review only

ACKNOWLEDGEMENTS

Author Contributions

PU had full access to all the data in the study and take responsibility for the integrity of the data and the accuracy of the data analysis. PT, TJ, TF, MJ, MS, DA, and PU developed the research design. PT, TJ, GB, AB, GE, JE, TF, MG, CJ, LL, MS, JS, SS, SZ, CÖ, DA and PU contributed to the acquisition, analysis, or interpretation of data. DA and PU drafted first draft of the manuscript. PT, TJ, GB, AB, GE, JE, TF, MG, CJ, LL, MS, JS, SS, SZ, CÖ, DA and PU critically reviewed and revised the manuscript for important intellectual content and approved the manuscript. PU performed the statistical analysis. TJ and PU obtained funding.

Funding/Support

The study was supported by a grant from the Swedish Heart-Lung Foundation. PU was supported by grants from the Swedish Heart-Lung Foundation and the Swedish Society for Medical Research. DPA was supported by grants from CIMED. The main funding body of The Swedish CArdioPulmonary bioImage Study (SCAPIS) is the Swedish Heart-Lung Foundation. The study is also funded by the Knut and Alice Wallenberg Foundation, the Swedish Research Council and VINNOVA (Sweden's Innovation agency) the University of Gothenburg and Sahlgrenska University Hospital, Karolinska Institutet and Stockholm county council, Linköping University and University Hospital, Lund University and Skåne University Hospital, Umeå University and University Hospital, Uppsala University and University Hospital.

Role of the Funder/Sponsor

For peer review only

The funding sources had no role in the design and conduct of the study; collection, management, analysis, and interpretation of the data; preparation, review, or approval of the manuscript; and decision to submit the manuscript for publication.

Conflict of Interest Disclosures

The authors have no conflict of interest to report.

Data sharing

No additional data available.

Ethical approval

The study was approved by the Ethical Review Board in Umeå, Sweden (reference numbers: 2010-228-31M and 2020-02668).

REFERENCES

1 Wu C, Chen X, Cai Y, *et al.* Risk Factors Associated With Acute Respiratory Distress Syndrome and Death in Patients With Coronavirus Disease 2019 Pneumonia in Wuhan, China. *JAMA Intern Med* Published Online First: 13 March 2020. doi:10.1001/jamainternmed.2020.0994

2 Docherty AB, Harrison EM, Green CA, *et al.* Features of 20 133 UK patients in hospital with covid-19 using the ISARIC WHO Clinical Characterisation Protocol: prospective observational cohort study. *BMJ* 2020;**369**:m1985. doi:10.1136/bmj.m1985

3 McGurnaghan SJ, Weir A, Bishop J, *et al.* Risks of and risk factors for COVID-19 disease in people with diabetes: a cohort study of the total population of Scotland. *Lancet Diabetes Endocrinol* Published Online First: 23 December 2020. doi:10.1016/S2213-8587(20)30405-8

For peer review only

4 Barron E, Bakhai C, Kar P, *et al.* Associations of type 1 and type 2 diabetes with COVID-19-related mortality in England: a whole-population study. *Lancet Diabetes Endocrinol* 2020;**8**:813–22. doi:10.1016/S2213-8587(20)30272-2

5 Reilev M, Kristensen KB, Pottegård A, *et al.* Characteristics and predictors of hospitalization and death in the first 11 122 cases with a positive RT-PCR test for SARS-CoV-2 in Denmark: a nationwide cohort. *Int J Epidemiol* 2020;**49**:1468–81. doi:10.1093/ije/dyaa140

6 Petrilli CM, Jones SA, Yang J, *et al.* Factors associated with hospital admission and critical illness among 5279 people with coronavirus disease 2019 in New York City: prospective cohort study. *BMJ* 2020;**369**:m1966. doi:10.1136/bmj.m1966

7 Clift AK, Coupland CAC, Keogh RH, *et al.* Living risk prediction algorithm (QCOVID) for risk of hospital admission and mortality from coronavirus 19 in adults: national derivation and validation cohort study. *BMJ* 2020;**371**:m3731. doi:10.1136/bmj.m3731

8 Hamer M, Gale CR, Kivimäki M, *et al.* Overweight, obesity, and risk of hospitalization for COVID-19: A community-based cohort study of adults in the United Kingdom. *Proc Natl Acad Sci U S A* 2020;**117**:21011–3. doi:10.1073/pnas.2011086117

9 Bergström G, Berglund G, Blomberg A, *et al.* The Swedish CArdioPulmonary BioImage Study: objectives and design. *J Intern Med* 2015;**278**:645–59. doi:10.1111/joim.12384

10 Rolfhamre P, Janson A, Arneborn M, *et al.* SmiNet-2: Description of an internet-based surveillance system for communicable diseases in Sweden. *Eurosurveillance* 2006;**11**:15–6. doi:10.2807/esm.11.05.00626-en

11 Ludvigsson JF. The first eight months of Sweden's COVID-19 strategy and the key actions and actors that were involved. *Acta Paediatr Int J Paediatr* 2020;**109**:2459–71. doi:10.1111/apa.15582

12 Drefahl S, Wallace M, Mussino E, *et al.* A population-based cohort study of socio-demographic risk factors for COVID-19 deaths in Sweden. *Nat Commun* 2020;**11**:5097. doi:10.1038/s41467-020-18926-3

13 Xie J, Zu Y, Alkhatib A, *et al.* Metabolic Syndrome and COVID-19 Mortality Among Adult Black Patients in New Orleans. *Diabetes Care* 2021;**44**:188–93. doi:10.2337/dc20-1714

14 Garg H, Khanna P. Covid and cholesterol (C&C): Something to worry about or much ado about nothing? *Trends Anaesth Crit Care* Published Online First: September 2020. doi:10.1016/j.tacc.2020.09.003

15 Hopkinson NS, Rossi N, El-Sayed_Moustafa J, *et al.* Current smoking and COVID-19 risk: results from a population symptom app in over 2.4 million people. *Thorax* 2021;:thoraxjnl-2020-216422. doi:10.1136/thoraxjnl-2020-216422

16 Wenzl T. Smoking and COVID-19 - A review of studies suggesting a protective effect of smoking against COVID-19. EU Sci. Hub. doi:10.2760/564217

For peer review only

17 Dillinger JG, Benmessaoud FA, Pezel T, *et al.* Coronary Artery Calcification and Complications in Patients With COVID-19. *JACC Cardiovasc Imaging* 2020;**13**:2468–70. doi:10.1016/j.jcmg.2020.07.004

18 Clark CE, McDonagh STJ, McManus RJ, *et al.* COVID-19 and hypertension: risks and management. A scientific statement on behalf of the British and Irish Hypertension Society. J. Hum. Hypertens. 2021;:1–4. doi:10.1038/s41371-020-00451-x

19 Ludvigsson JF. The first eight months of Sweden's COVID-19 strategy and the key actions and actors that were involved. *Acta Paediatr* 2020;:apa.15582. doi:10.1111/apa.15582

For peer review only

Supplementary Table 1 Definitions and categorization of selected variables.

Variable	Categorization	n (%) missing
**Sociodemographic information**		
Age	Continuous in years	0 (0)
Sex	1. Women<br>2. Men	0 (0)
Place of birth	1. Not born in Sweden<br>2. Born in Sweden	811 (2.7)
Education	1. High school or less<br>2. Vocational education<br>3. University	845 (2.8)
**Cardiometabolic risk factors**		
Diabetes status	1. Normoglycemia<br>2. Prediabetes (fasting glucose [6.1-6.9 mmol/L or glycated hemoglobin ≥42 mmol/mol and <48 mmol/mol])<br>3. Diabetes diagnosis by physician (self-reported in questionnaire) or glycated hemoglobin ≥48 mmol/mol.	145 (0.5)
Glycated hemoglobin	Continuous in mmol/mol	152 (0.5)
Body mass index	Continuous in kg/m^2	2 (0)
Weight status	1. Normal weight<br>2. Overweight<br>3. Obesity	2 (0)
Waist-hip ratio	Continuous	1567 (5.2)
Systolic blood pressure	Continuous in mmHg	151 (0.5)
Diastolic blood pressure	Continuous in mmHg	153 (0.5)
Blood pressure level	Level as measured at inclusion in SCAPIS.<br>1. Normotensive (systolic blood pressure <140 mmHg and diastolic blood pressure <90 mmHg)<br>2. Grade 1 hypertension (Systolic blood pressure ≥140 mmHg and <160 mmHg or diastolic blood pressure ≥90 mmHg and <100 mmHg)<br>3. Grade 2 hypertension (systolic blood pressure ≥160 mmHg or diastolic blood pressure ≥100 mmHg).	153 (0.5)
Current smoking	Self-reported in questionnaire.<br>1. No<br>2. Yes	954 (3.2)
Time spent sedentary per day	Self-reported in questionnaire.<br>Continuous (hours per day)	10441 (34.9)
Coronary artery calcium score by computer tomography	Continuous	1197 (4)
Total cholesterol	Continuous in mmol/L	82 (0.3)
Low-density lipoprotein (LDL) cholesterol	Continuous in mmol/L	218 (0.7)
HDL cholesterol	Continuous in mmol/L	85 (0.3)
Creatinine	Continuous in mikromol/L	61 (0.2)

a. n (%) missing values out of the total study population (n=29,955)

For peer review only

Supplementary Table 2 Characteristics of SCAPIS participants by their status of laboratory-confirmed diagnosis of Covid-19 between January 31 and September 12, 2020. Numbers are shown in n (%) unless otherwise indicated.

	No laboratory-confirmed diagnosis of Covid-19	Laboratory-confirmed diagnosis of Covid-19	
		Hospitalization or death due to Covid-19	
n		No	Yes
	29656	230	69
Age, mean (SD)	61.2 (4.5)	59.9 (4.0)	61.9 (4.8)
Men	14408 (48.6)	75 (32.6)	52 (75.4)
Born outside of Sweden	4676 (16.2)	49 (21.7)	22 (32.4)
Education			
High School or less	2686 (9.3)	14 (6.2)	12 (17.9)
Vocational	13120 (45.5)	98 (43.4)	31 (46.3)
University	13011 (45.2)	114 (50.4)	24 (35.8)
Weight status			
Normal weight	10643 (35.9)	95 (41.3)	8 (11.6)
Overweight	12723 (42.9)	91 (39.6)	36 (52.2)
Obesity	6288 (21.2)	44 (19.1)	25 (36.2)
Body mass index in kg/m^2, mean (SD)	27.0 (4.5)	26.5 (4.2)	30 (5.3)
Diabetes status			
Normoglycemia	22582 (76.5)	192 (83.5)	32 (46.4)
Prediabetes	4701 (15.9)	26 (11.3)	20 (29)
Diabetes	2228 (7.5)	12 (5.2)	17 (24.6)
Waist-hip ratio, mean (SD)	0.9 (0.1)	0.90 (0.08)	0.98 (0.09)
Current smoking	3768 (13.1)	17 (7.7)	6 (9.0)

For peer review only

Sedentary time per day in hours, mean (SD)	6.9 (3.5)	6.1 (3.4)	8.3 (4.5)
Blood pressure level			
Normotensive	23100 (78.3)	185 (81.1)	50 (72.5)
Grade 1 hypertension	5093 (17.3)	36 (15.8)	12 (17.4)
Grade 2 hypertension	1312 (4.4)	7 (3.1)	7 (10.1)
Systolic blood pressure, mean (SD)	125.9 (17.0)	124.2 (16.9)	133.5 (19.5)
Diastolic blood pressure, mean (SD)	77.5 (10.5)	76.6 (10.0)	81 (12.4)
Triglycerides in mmol/L, mean (SD)	1.2 (0.8)	1.2 (0.8)	1.6 (1.1)
HDL in mmol/L, mean (SD)	1.6 (0.5)	1.7 (0.5)	1.3 (0.4)
Total cholesterol in mmol/L, mean (SD)	5.5 (1.1)	5.6 (1.0)	5.3 (1.0)
LDL cholesterol in mmol/L, mean (SD)	3.4 (1.0)	3.6 (0.9)	3.3 (0.9)
Glycated hemoglobin in mmol/mol, mean (SD)	36.6 (6.5)	35.9 (5.6)	39.7 (9.3)
Creatinine in μmol/L, mean (SD)	77.7 (16.4)	74.6 (14.4)	86.2 (37.9)
Coronary artery calcium score, mean (SD)	61.8 (230.1)	24.4 (119.9)	90.2 (199.6)

For peer review only

STROBE Statement—Checklist of items that should be included in reports of ***cohort studies***

	Item No	Recommendation	Page
**Title and abstract**	1	(*a*) Indicate the study's design with a commonly used term in the title or the abstract	Title and abstract
		(*b*) Provide in the abstract an informative and balanced summary of what was done and what was found	Abstract
**Introduction**			
Background/rationale	2	Explain the scientific background and rationale for the investigation being reported	p.8
Objectives	3	State specific objectives, including any prespecified hypotheses	p.8
**Methods**			
Study design	4	Present key elements of study design early in the paper	p.8-10
Setting	5	Describe the setting, locations, and relevant dates, including periods of recruitment, exposure, follow-up, and data collection	p.8-10
Participants	6	(*a*) Give the eligibility criteria, and the sources and methods of selection of participants. Describe methods of follow-up	p. 10
		(*b*) For matched studies, give matching criteria and number of exposed and unexposed	
Variables	7	Clearly define all outcomes, exposures, predictors, potential confounders, and effect modifiers. Give diagnostic criteria, if applicable	p. 8-10 Supplementary Material (Supplementary Table 1).
Data sources/ measurement	8*	For each variable of interest, give sources of data and details of methods of assessment (measurement). Describe comparability of assessment methods if there is more than one group	8-10, Supplementary Table 1.
Bias	9	Describe any efforts to address potential sources of bias	p. 8-10
Study size	10	Explain how the study size was arrived at	p.10
Quantitative variables	11	Explain how quantitative variables were handled in the analyses. If applicable, describe which groupings were chosen and why	p.9-10, Supplementary Table 1
Statistical methods	12	(*a*) Describe all statistical methods, including those used to control for confounding	p.10-11
		(*b*) Describe any methods used to examine subgroups and interactions	p.10-11
		(*c*) Explain how missing data were addressed	p.10
		(*d*) If applicable, explain how loss to follow-up was addressed	NA
		(*e*) Describe any sensitivity analyses	NA
**Results**			
Participants	13*	(a) Report numbers of individuals at each stage of study—eg numbers potentially eligible, examined for eligibility, confirmed eligible, included in the study, completing follow-up, and analysed	p.10
		(b) Give reasons for non-participation at each stage	p.10
		(c) Consider use of a flow diagram	NA
Descriptive data	14*	(a) Give characteristics of study participants (eg demographic, clinical, social) and information on exposures and potential	Table 1

For peer review only - http://bmjopen.bmj.com/site/about/guidelines.xhtml

		confounders	
		(b) Indicate number of participants with missing data for each variable of interest	Table 1
		(c) Summarise follow-up time (eg, average and total amount)	p.10
Outcome data	15*	Report numbers of outcome events or summary measures over time	p.11
Main results	16	(*a*) Give unadjusted estimates and, if applicable, confounder-adjusted estimates and their precision (eg, 95% confidence interval). Make clear which confounders were adjusted for and why they were included	p.10-13, Table 1, Table 2
		(*b*) Report category boundaries when continuous variables were categorized	Supplementary Table 1
		(*c*) If relevant, consider translating estimates of relative risk into absolute risk for a meaningful time period	Not deemed relevant
Other analyses	17	Report other analyses done—eg analyses of subgroups and interactions, and sensitivity analyses	p. 10-13
**Discussion**			
Key results	18	Summarise key results with reference to study objectives	p.15
Limitations	19	Discuss limitations of the study, taking into account sources of potential bias or imprecision. Discuss both direction and magnitude of any potential bias	p.16
Interpretation	20	Give a cautious overall interpretation of results considering objectives, limitations, multiplicity of analyses, results from similar studies, and other relevant evidence	p.15
Generalisability	21	Discuss the generalisability (external validity) of the study results	p.15-16
**Other information**			
Funding	22	Give the source of funding and the role of the funders for the present study and, if applicable, for the original study on which the present article is based	p.17

*Give information separately for exposed and unexposed groups.

Note: An Explanation and Elaboration article discusses each checklist item and gives methodological background and published examples of transparent reporting. The STROBE checklist is best used in conjunction with this article (freely available on the Web sites of PLoS Medicine at http://www.plosmedicine.org/, Annals of Internal Medicine at http://www.annals.org/, and Epidemiology at http://www.epidem.com/). Information on the STROBE Initiative is available at http://www.strobe-statement.org.

For peer review only

BMJ Open

Association of cardiometabolic risk factors with hospitalization or death due to COVID-19: population-based cohort study in Sweden (SCAPIS)

Journal:	*BMJ Open*
Manuscript ID	bmjopen-2021-051359.R2
Article Type:	Original research
Date Submitted by the Author:	28-Jul-2021
Complete List of Authors:	Tornhammar, Per; Karolinska University Hospital, Functional Area of Emergency Medicine<br>Jernberg, Tomas; Karolinska Institutet, Department of Clinical Sciences, Danderyd University Hospital<br>Bergström, Göran; Sahlgrenska University Hospital, Clinical Physiology; University of Gothenburg Sahlgrenska Academy, Department of Molecular and Clinical Medicine<br>Blomberg, Anders; Umeå University, Department of Public Health and Clinical Medicine, Section of Medicine<br>Engström, Gunnar; Lund University, Department of Clinical Science in Malmö<br>Engvall, Jan; Linköping University, CMIV, Centre of Medical Image Science and Visualization; Linköping University, Department of Clinical Physiology, and Department of Health, Medicine and Caring Sciences,<br>Fall, Tove; Uppsala University, Medical Sciences<br>Gisslén, Magnus; University of Gothenburg Sahlgrenska Academy, Department of Infectious Diseases, Institute of Biomedicine; Sahlgrenska University Hospital, Department of Infectious Diseases, Region Västra Götaland<br>Janson, Christer; Uppsala University, Dep om Medical Sciences: Respiratory Medicine<br>Lind, Lars; Uppsala University, Department of Medical Sciences, Clinical Epidemiology<br>Skold, Magnus; Karolinska Institute, Respiratory Medicine; Karolinska University Hospital, Respiratory Medicine and Allergy<br>Sundström, Johan; Uppsala University, Department of Medical Sciences, Clinical Epidemiology; University of New South Wales, The George Institute for Global Health<br>Söderberg, Stefan ; Umeå University, Department of Public Health and Clinical Medicine, Section of Medicine<br>Zaigham, Suneela; Lund University, Department of Clinical Science in Malmö<br>Östgren, Carl Johan; Linköping University, Department of Health, Medicine and Caring Sciences<br>Andersson, Daniel Peter; Karolinska University Hospital, Department of Medicine Huddinge H7, Karolinska Institutet<br>Ueda, Peter; Karolinska Institutet, Clinical Epidemiology Division, Department of Medicine, Solna

**Primary Subject Heading**:	Epidemiology
Secondary Subject Heading:	Cardiovascular medicine, Public health
Keywords:	COVID-19, Epidemiology < INFECTIOUS DISEASES, Cardiac Epidemiology < CARDIOLOGY

SCHOLARONE™ Manuscripts

I, the Submitting Author has the right to grant and does grant on behalf of all authors of the Work (as defined in the below author licence), an exclusive licence and/or a non-exclusive licence for contributions from authors who are: i) UK Crown employees; ii) where BMJ has agreed a CC-BY licence shall apply, and/or iii) in accordance with the terms applicable for US Federal Government officers or employees acting as part of their official duties; on a worldwide, perpetual, irrevocable, royalty-free basis to BMJ Publishing Group Ltd ("BMJ") its licensees and where the relevant Journal is co-owned by BMJ to the co-owners of the Journal, to publish the Work in this journal and any other BMJ products and to exploit all rights, as set out in our licence.

The Submitting Author accepts and understands that any supply made under these terms is made by BMJ to the Submitting Author unless you are acting as an employee on behalf of your employer or a postgraduate student of an affiliated institution which is paying any applicable article publishing charge ("APC") for Open Access articles. Where the Submitting Author wishes to make the Work available on an Open Access basis (and intends to pay the relevant APC), the terms of reuse of such Open Access shall be governed by a Creative Commons licence – details of these licences and which Creative Commons licence will apply to this Work are set out in our licence referred to above.

Other than as permitted in any relevant BMJ Author's Self Archiving Policies, I confirm this Work has not been accepted for publication elsewhere, is not being considered for publication elsewhere and does not duplicate material already published. I confirm all authors consent to publication of this Work and authorise the granting of this licence.

For peer review only

Association of cardiometabolic risk factors with hospitalization or death due to COVID-19: population-based cohort study in Sweden (SCAPIS)

Per Tornhammar[1], Tomas Jernberg[2], Göran Bergström[3,4], Anders Blomberg[5], Gunnar Engström[6], Jan Engvall[7,8], Tove Fall[9], Magnus Gisslen[10,11], Christer Janson[12], Lars Lind[13], Magnus Sköld[14,15], Johan Sundström[13,16], Stefan Söderberg[5], Suneela Zaigham[6], Carl Johan Östgren[17], Daniel Peter Andersson[18]*, Peter Ueda[19]*

*Shared last authorship

[1] Functional Area of Emergency Medicine, Karolinska University Hospital, Huddinge, Stockholm, Sweden.

[2] Department of Clinical Sciences, Danderyd Hospital, Karolinska Institutet, Stockholm, Sweden.

[3] Department of Molecular and Clinical Medicine, Sahlgrenska Academy, University of Gothenburg, Gothenburg, Sweden.

[4] Clinical Physiology, Sahlgrenska University Hospital, Gothenburg, Sweden.

[5] Department of Public Health and Clinical Medicine, Section of Medicine, Umeå University, Umeå, Sweden.

[6] Department of Clinical Science in Malmö, Lund University, 20502 Malmö, Sweden.

[7] CMIV, Centre of Medical Image Science and Visualization, Linköping University, Linköping, Sweden.

[8] Department of Clinical Physiology, and Department of Health, Medicine and Caring Sciences, Linköping University, Linköping, Sweden.

[9] Department of Medical Sciences, Molecular Epidemiology and Science for Life Laboratory, Uppsala University, Uppsala, Sweden.

[10] Department of Infectious Diseases, Institute of Biomedicine, Sahlgrenska Academy, University of Gothenburg, Gothenburg, Sweden.

[11] Department of Infectious Diseases, Region Västra Götaland, Sahlgrenska University Hospital, Gothenburg, Sweden.

[12] Department of Medical Sciences Respiratory, Allergy and Sleep Research, Uppsala University, Uppsala, Sweden.

[13] Department of Medical Sciences, Clinical Epidemiology, Uppsala University, Uppsala, Sweden.

For peer review only

[14] Department of Respiratory Medicine and Allergy, Karolinska University Hospital Solna, Stockholm, Sweden.

[15] Respiratory Medicine Unit, Department of Medicine Solna and Center for Molecular Medicine, Karolinska Institutet, Stockholm, Sweden.

[16] The George Institute for Global Health, University of New South Wales, Sydney, Australia.

[17] Department of Health, Medicine and Caring Sciences, Linköping University, Linköping, Sweden.

[18] Department of Medicine Huddinge H7, Karolinska Institutet, Karolinska University Hospital, Stockholm, Sweden.

[19] Clinical Epidemiology Division, Department of Medicine, Solna, Karolinska Institutet, Stockholm, Sweden.

Word count: 1707

Word count abstract: 271

Tables: 2 Tables

Online supplement: 2 Supplementary Tables

Correspondence: Peter Ueda, MD, PhD. Clinical Epidemiology Division, Department of Medicine, Solna, Eugeniahemmet, T2, Karolinska University Hospital, 17176, Stockholm, Sweden. Email: peter.ueda@ki.se

For peer review only

Abstract

Objective

To assess the association of cardiometabolic risk factors with hospitalization or death due to Covid-19 in the general population.

Design, setting and participants

Swedish population-based cohort including 29,955 participants.

Exposures

Cardiometabolic risk factors assessed between 2014 and 2018.

Main outcome measures

Hospitalization or death due to Covid-19, as registered in nationwide registers from January 31 2020 through September 12, 2020. Associations of cardiometabolic risk factors with the outcome were assessed using logistic regression adjusted for age, sex, birthplace, and education.

Results

Mean [SD] age was 61.2 [4.5] and 51.5% were women. 69 participants experienced hospitalization or death due to Covid-19. Examples of statistically significant associations between baseline factors and subsequent hospitalization or death due to Covid-19 included overweight (adjusted odds ratio [aOR] vs normal weight 2.73 [95% CI 1.25-5.94]), obesity (aOR vs normal weight 4.09 [95% CI 1.82-9.18]), prediabetes (aOR vs normoglycemia 2.56 [95% CI 1.44-4.55]), diabetes (aOR vs normoglycemia 3.96 [95% CI 2.13-7.36]), sedentary time (aOR per hour/day increase 1.10 [95% CI 1.02-1.17]), grade 2 hypertension (aOR vs normotension 2.44 [95% CI 1.10-5.44]) and high density lipoprotein cholesterol (aOR per mmol/L increase 0.33 [95% CI 0.17-0.65]).

For peer review only

Statistically significant associations were not observed for grade 1 hypertension (aOR vs normotension 1.03 [95% CI 0.55-1.96]), current smoking (aOR 0.56 [95% CI 0.24-1.30]), total cholesterol (aOR per mmol/L increase 0.90 [95% CI 0.71-1.13]), low density lipoprotein cholesterol (aOR per mmol/L increase 0.90 [95% CI 0.69-1.15]) and coronary artery calcium score (aOR per 10 units increase 1.00 [95% CI 0.99-1.01]).

Conclusions

In a large population-based sample from the general population, several cardiometabolic risk factors were associated with hospitalization or death due to Covid-19.

For peer review only

Strengths and limitations of this study

- This study used data on cardiometabolic risk factors measured between 2014 and 2018 in a population-based cohort of over 30,000 participants and assessed their association with hospitalization or death due to Covid-19 during the first wave of the pandemic.
- Few previous studies have used population-based samples from the general population to assess the relationship between cardiometabolic risk factors and outcomes in Covid-19.
- As we could not capture all cases of Covid-19 which did not lead to hospitalization or death, we could not assess to what extent the observed associations may reflect the relationship with exposure to SARS-CoV 2 as compared with the risk of hospitalization or death due to Covid-19 among those who have been exposed to the virus.

For peer review only

Identification of individuals at risk of worse outcomes in Covid-19 may inform risk management decisions to mitigate exposure and to prioritize vaccination. Several studies have shown an that cardiometabolic risk factors are associated with a higher risk of adverse outcomes in Covid-19. While many of these studies have been performed in selected populations such as patients hospitalized with Covid-19[1,2] or patients with certain diagnoses,[3,4] fewer studies have been based on data from the general population.[5–8]

The Swedish Cardiopulmonary bioimage study (SCAPIS)[9] is a population-based cohort of approximately 30,000 men and women who were extensively characterized with respect to cardiometabolic risk factors and function at the age of 50-64 years during the years preceding the Covid-19 outbreak (2014 through 2018). We used data from SCAPIS to assess the association of cardiometabolic risk factors with risk of hospitalization or death due to Covid-19 during the first wave of the pandemic.

METHODS

Data sources

The SCAPIS cohort, described in detail elsewhere,[9] is a population-based cohort conducted at six Swedish university hospitals in Gothenburg, Linköping, Malmö/Lund, Stockholm, Umeå and Uppsala, with each site recruiting participants from corresponding municipality areas. From 2014 through 2018, over 30,000 participants aged 50-64 years underwent examinations of cardiopulmonary risk factors and provided information regarding lifestyle factors and socioeconomic conditions. Using

For peer review only

the personal identity number, we linked SCAPIS data to nationwide administrative and health registers. From the National Patient Register, which comprises physician-assigned diagnoses based on the International Classification of Diseases, 10th Revision, Swedish Edition (ICD-10-SE) for all hospital admissions in Sweden, we obtained information about hospitalization for Covid-19. From the Cause of Death register, we obtained data about vital status, and date and cause of death. From SmiNet,[10] a reporting system for infectious diseases administered by the Public Health Agency, we obtained information about laboratory-confirmed cases of Covid-19, including those not leading to hospitalization or death.

Outcomes, exposures, and study period

The main outcome was a composite of hospitalization due to Covid-19 and death due to Covid-19. Hospitalization for Covid-19 was defined as hospital admission with laboratory-confirmed Covid-19 as the primary diagnosis (ICD-10-SE code U07.1 [Covid-19, virus identified]).[11] Death due to Covid-19 was defined as death where U07.1 was specified as the underlying cause of death. Follow-up was from January 31, 2020 (the first laboratory-confirmed case of Covid-19 in Sweden) through September 12, 2020.

We included variables in the SCAPIS dataset, (described in detail in Supplementary Table 1), that we hypothesized could be associated with hospitalization or death due to Covid-19: these included sociodemographic variables (age, sex, place of birth and education) and cardiometabolic risk factors: diabetes status (normoglycemia, prediabetes, diabetes); weight status (normal [body mass index [BMI] <25 kg/m^2], overweight [BMI ≥25 to <30 kg/m^2], obesity [BMI ≥30 kg/m^2]); current smoking, waist-hip ratio, blood pressure status (normotension, grade 1 hypertension, grade 2

hypertension); systolic and diastolic blood pressure; coronary artery calcium score; total cholesterol; low density lipoprotein (LDL) cholesterol; high density lipoprotein (HDL) cholesterol; glycated hemoglobin; and creatinine. Few (≤2%) participants had established cardiovascular disease, including coronary heart disease, stroke and heart failure; therefore, we did not assess these variables.

Study population

We included all 30,154 participants in SCAPIS. We excluded 2 participants who had missing data on vital status and 197 participants who died before January 31, 2020. The study population included 29,955 participants.

Statistical analysis

Analyses were performed in Stata 16.0. We described study participants with respect to the selected variables, separately among those who did not experience hospitalization or death due to Covid-19 and among those who did and assessed differences between the groups using the Chi-squared test for categorical variables and the t-test for continuous variables. For each investigated variable separately, we used logistic regression to assess its association with hospitalization or death due to Covid-19, adjusting for sociodemographic variables including age (continuous variable), sex, place of birth (outside of Sweden vs Sweden) and education (high school or less, vocational, university).[12] Analyses were performed among those with complete data for all variables included in the model. The proportion of missing data for the analyzed variables are shown in Supplementary Table 1 and ranged between 0 and 5% (0 to 8% in the main analyses using those with complete data on the variables assessed and adjusted for); the exception was time spent sedentary per day for which 34.9% of the

For peer review only - http://bmjopen.bmj.com/site/about/guidelines.xhtml

participants had missing data. In post-hoc sensitivity analyses, we performed the main analyses with more than 5% missing data (those assessing waist-hip ratio, time spent sedentary per day and coronary artery calcium score) using multiple imputation (10 imputed datasets) created with chained equations.

The relationship between the investigated variables and hospitalization or death due to Covid-19 may not only represent the risk of worse Covid-19 outcomes in individuals exposed to SARS-CoV-2 but also the risk of exposure to SARS-CoV-2. We therefore performed an additional analysis restricted to the subgroup of participants (n=299) with a laboratory-confirmed Covid-19 diagnosis as recorded in SmiNet, the National Patient Register or the Cause of Death Register (i.e., participants with a laboratory-confirmed of Covid-19, including both those who did and did not experience hospitalization or death due to Covid-19). In this subgroup, we used logistic regression to assess the association of each of the cardiometabolic risk factors (separately) with hospitalization or death due to Covid-19. Odds ratios whose 95% CI did not overlap 1 were considered as statistically significant. The Swedish Ethical Review Authority approved the study.

Patient involvement

No patients were involved in setting the research question, nor in the design, conduct, or interpretation of the study. The study findings are planned to be disseminated through the SCAPIS website.

RESULTS

For peer review only

Characteristics of the 29,955 study participants are shown in Table 1. 14,535 (48.5%) were men. Mean (SD) age was 61.2 (4.5) years. 69 (0.2%) of the participants were hospitalized or died due to Covid-19. Those who experienced (n=69) vs did not experience (n=29,866) hospitalization or death due to Covid-19 were slightly older (61.9 years vs 61.2 years) and more likely to be men (75.4% vs 48.5%), born outside of Sweden (32.4% vs 16.3%) and to have education of high school or less (17.9% vs 9.3%).

Table 1 Characteristics of SCAPIS participants by experience of hospitalization or death due to Covid-19 between January 31 and September 12, 2020. Numbers are shown in n (%) unless otherwise indicated.

	Hospitalization or death due to Covid-19		
	No (n=29,866)	Yes (n=69)	P-value
Age, mean (SD)	61.2 (4.5)	61.9 (4.8)	0.226
Men	14483 (48.5)	52 (75.4)	<0.001
Born outside of Sweden	4725 (16.3)	22 (32.4)	<0.001
Education			
High School or less	2700 (9.3)	12 (17.9)	
Vocational	13218 (45.5)	31 (46.3)	
University	13125 (45.2)	24 (35.8)	0.036
Weight status			
Normal weight	10738 (35.9)	8 (11.6)	
Overweight	12814 (42.9)	36 (52.2)	
Obesity	6332 (21.2)	25 (36.2)	<0.001
Body mass index in kg/m^2, mean (SD)	27.0 (4.5)	30.0 (5.3)	<0.001
Diabetes status			
Normoglycemia	22774 (76.6)	32 (46.4)	
Prediabetes	4727 (15.9)	20 (29.0)	
Diabetes	2240 (7.5)	17 (24.6)	<0.001
Waist-hip ratio, mean (SD)	0.92 (0.09)	0.98 (0.09)	<0.001
Current smoking	3785 (13.1)	6 (9)	0.317
Sedentary time per day in hours, mean (SD)	6.9 (3.5)	8.3 (4.5)	0.009
Blood pressure measurement			
Normotensive	23285 (78.3)	50 (72.5)	
Grade 1 hypertension	5129 (17.3)	12 (17.4)	
Grade 2 hypertension	1319 (4.4)	7 (10.1)	0.069
Systolic blood pressure, mean (SD)	125.9 (17.0)	133.5 (19.5)	<0.001
Diastolic blood pressure, mean (SD)	77.5 (10.5)	81.0 (12.4)	0.006
Triglycerides in mmol/L, mean (SD)	1.2 (0.8)	1.6 (1.1)	<0.001
HDL in mmol/L, mean (SD)	1.6 (0.5)	1.3 (0.4)	<0.001
Total cholesterol in mmol/L, mean (SD)	5.5 (1.1)	5.3 (1.0)	0.068
LDL cholesterol in mmol/L, mean (SD)	3.4 (1.0)	3.3 (0.9)	0.360

For peer review only

Glycated hemoglobin in mmol/mol, mean (SD)	36.6 (6.4)	39.7 (9.3)	<0.001
Creatinine in μmol/L, mean (SD)	77.7 (16.4)	86.2 (37.9)	<0.001
Coronary artery calcium score, mean (SD)	61.6 (229.5)	90.2 (199.6)	0.053[a]

[a] Because 17016 of the participants had a score of 0, the p-value calculated using the Chi-squared with five categories: 0 and quartiles of score among participants with a score of >0.

The results of the logistic regression assessing the association between each of the selected cardiometabolic risk factors and hospitalization or death due to Covid-19 (adjusted for age, sex, place of birth and education) are shown in Table 2. Significant associations were observed for overweight and obesity, higher body mass index, prediabetes, diabetes, higher waist-hip ratio, more time spent sedentary per day, grade 2 hypertension, as well as higher systolic blood pressure, diastolic blood pressure, triglycerides and glycated hemoglobin, and lower HDL cholesterol. Significant associations were not observed for grade 1 hypertension, current smoking, total cholesterol, LDL cholesterol, creatinine and coronary artery calcium score. In the post-hoc sensitivity analyses using multiple imputation for logistic regression models with over 5% missing data, the results were similar to those in the main analyses: waist hip ratio (odds ratio per SD increase 1.54 [95% CI 1.20 to 1.98], time spent sedentary per day (odds ratio per hour increase 1.09 [95% CI 1.01 to 1.18] and coronary artery calcium score (odds ratio per 10 units increase 1.00 [95% CI 0.99 to 1.01])

For peer review only

Table 2 Odds ratios (95% CI)[a], adjusted for age, sex, place of birth and education, for the association of selected variables with hospitalization or death due to Covid-19 in the main and additional analyses.

	Main analysis (n=29,955)	Additional analysis restricted to those with laboratory-confirmed Covid-19 diagnosis (n=299)
Age per year	1.03 (0.97 - 1.08)	1.11 (1.04 - 1.20)
Men	3.39 (1.93 - 5.95)	6.62 (3.45 - 12.71)
Born outside of Sweden	2.40 (1.43 - 4.03)	1.59 (0.80 - 3.18)
Education [b]		
High School or less	1.00 (ref)	1.00 (ref)
Vocational	0.58 (0.30 - 1.14)	0.41 (0.16 - 1.09)
University	0.49 (0.24 - 0.99)	0.26 (0.10 - 0.70)
Weight status		
Normal weight	1.00 (ref)	1.00 (ref)
Overweight	2.73 (1.25 - 5.94)	3.41 (1.41 - 8.27)
Obesity	4.09 (1.82 - 9.18)	4.86 (1.86 - 12.71)
Body mass index per 5 kg/m^2 increase	1.77 (1.43 - 2.19)	2.05 (1.44 - 2.92)
Diabetes status		
Normoglycemia	1.00 (ref)	1.00 (ref)
Prediabetes	2.56 (1.44 - 4.55)	3.72 (1.68 - 8.28)
Diabetes	3.96 (2.13 - 7.36)	5.12 (1.95 - 13.42)
Waist-hip ratio per SD increase	1.55 (1.20 - 2.00)	1.92 (1.20 - 3.06)
Sedentary time per day per hour increase	1.10 (1.02 - 1.17)	1.13 (1.02 - 1.26)
Blood pressure measurement		
Normotensive	1.00 (ref)	1.00 (ref)
Grade 1 hypertension	1.03 (0.55 - 1.96)	0.95 (0.41 - 2.17)
Grade 2 hypertension	2.44 (1.10 - 5.44)	4.18 (1.07 - 16.32)
Systolic blood pressure per 10 mmHg increase	1.22 (1.07 - 1.40)	1.21 (1.01 - 1.44)
Diastolic blood pressure per 10 mmHg increase	1.31 (1.05 - 1.64)	1.30 (0.97 - 1.73)
Current smoking	0.56 (0.24 - 1.30)	0.87 (0.28 - 2.71)
Triglycerides per mmol/L increase	1.10 (1.00 - 1.21)	1.34 (0.98 - 1.85)
HDL per mmol/L increase	0.33 (0.17 - 0.65)	0.26 (0.10 - 0.66)
Total cholesterol per mmol/L increase	0.90 (0.71 - 1.13)	0.80 (0.59 - 1.10)
LDL cholesterol per mmol/L increase	0.90 (0.69 - 1.15)	0.83 (0.59 - 1.17)
Glycated hemoglobin per mmol/mol increase	1.03 (1.01 - 1.05)	1.04 (1.00 - 1.09)
Creatinine per 10 µmol/L increase	1.05 (1.00 - 1.10)	1.04 (0.88 - 1.23)
Coronary artery calcium score per 10 units increase	1.00 (0.99 - 1.01)	1.00 (0.98 - 1.02)

[a] Reference group for binary variables (Yes/No) is "No".

[b] Adjusted for sex and place of birth.

For peer review only

Population characteristics for the additional analysis including the 299 participants with laboratory confirmed Covid-19 are shown in Supplementary Table 2. Compared to the total study population, those with a laboratory-confirmed diagnosis of Covid-19 were less likely to be men and to be current smokers and more likely to be born outside of Sweden. The results of the logistic regression analyses are shown in Table 2. The findings were largely similar to those of the main analysis.

Discussion

We assessed the association of cardiometabolic risk factors with risk of hospitalization or death due to Covid-19 during the first wave of the pandemic in a Swedish population-based cohort with nearly 30,000 participants aged 52-72 years in 2020.

In analyses adjusted for age, sex, place of birth and education, we found that several cardiometabolic risk factors were associated with an increased risk of hospitalization or death due to Covid-19. Significant associations were found for metabolic risk factors, including overweight, obesity, prediabetes, diabetes and higher waist-hip ratio and glycated hemoglobin; findings that are in line with previous studies on these or related risk factors.[7,8,13] While significant associations were observed for grade 2 hypertension and for systolic and diastolic blood pressure when analyzed as continuous variables, grade 1 hypertension was not associated with an increased risk. Moreover, while lower HDL cholesterol and higher triglycerides were associated with hospitalization or death due to Covid-19, such associations were not observed for LDL cholesterol or total cholesterol. Associations with hospitalization or death due to Covid-19 were also not observed for current smoking and a higher coronary artery calcium

For peer review only

score. Previous studies on the association of smoking, hypertension, lipid levels and coronary artery calcium score with Covid-19 outcomes have yielded mixed findings.[8,14–18]

Strengths of our study include the use of a large sample from the general population who had undergone assessment of cardiometabolic risk factors in the years preceding the pandemic. Our study has limitations. Although the SCAPIS cohort includes detailed data on cardiometabolic risk factors from a large number of participants, the limited number of Covid-19 cases leading to hospitalization or death resulted in wide confidence intervals for some of the analyses. Moreover, during the first months of the pandemic, laboratory testing for Covid-19 was not widely performed and predominantly focused on healthcare professionals and hospitalized patients.[19] As such, although the findings of our analyses restricted to those with a laboratory-confirmed Covid-19 diagnosis were similar to those of our main analyses (although the analyses were based on a small sample of 299 participants), we could not assess to what extent the observed associations may reflect the relationship with exposure to SARS-CoV 2 as compared with the risk of hospitalization or death due to Covid-19 among those who have been exposed to the virus. Updated analyses may be performed as data on broader testing for Covid-19 and more cases of hospitalization or death due to Covid-19 become available. Finally, the study included individuals in a limited age range.

Conclusion

For peer review only - http://bmjopen.bmj.com/site/about/guidelines.xhtml

In this study large population-based cohort from the general population, several cardiometabolic risk factors were associated with hospitalization or death due to Covid-19.

For peer review only

ACKNOWLEDGEMENTS

Author Contributions

PU had full access to all the data in the study and take responsibility for the integrity of the data and the accuracy of the data analysis. PT, TJ, TF, CJ, MS, DA, and PU developed the research design. PT, TJ, GB, AB, GE, JE, TF, MG, CJ, LL, MS, JS, SS, SZ, CÖ, DA and PU contributed to the acquisition, analysis, or interpretation of data. DA and PU drafted first draft of the manuscript. PT, TJ, GB, AB, GE, JE, TF, MG, CJ, LL, MS, JS, SS, SZ, CÖ, DA and PU critically reviewed and revised the manuscript for important intellectual content and approved the manuscript. PU performed the statistical analysis. TJ and PU obtained funding.

Funding/Support

The study was supported by a grant from the Swedish Heart-Lung Foundation (grant number 20200491). PU was supported by grants from the Swedish Heart-Lung Foundation and the Swedish Society for Medical Research. DPA was supported by grants from CIMED (grant number 20180855). The main funding body of The Swedish CArdioPulmonary bioImage Study (SCAPIS) is the Swedish Heart-Lung Foundation. The study is also funded by the Knut and Alice Wallenberg Foundation, the Swedish Research Council and VINNOVA (Sweden's Innovation agency) the University of Gothenburg and Sahlgrenska University Hospital, Karolinska Institutet and Stockholm county council, Linköping University and University Hospital, Lund University and Skåne University Hospital, Umeå University and University Hospital, Uppsala University and University Hospital.

For peer review only

Role of the Funder/Sponsor

The funding sources had no role in the design and conduct of the study; collection, management, analysis, and interpretation of the data; preparation, review, or approval of the manuscript; and decision to submit the manuscript for publication.

Conflict of Interest Disclosures

The authors have no conflict of interest to report.

Data sharing

No additional data available.

Ethical approval

The study was approved by the Ethical Review Board in Umeå, Sweden (reference numbers: 2010-228-31M and 2020-02668).

REFERENCES

1 Wu C, Chen X, Cai Y, *et al.* Risk Factors Associated With Acute Respiratory Distress Syndrome and Death in Patients With Coronavirus Disease 2019 Pneumonia in Wuhan, China. *JAMA Intern Med* Published Online First: 13 March 2020. doi:10.1001/jamainternmed.2020.0994

2 Docherty AB, Harrison EM, Green CA, *et al.* Features of 20 133 UK patients in hospital with covid-19 using the ISARIC WHO Clinical Characterisation Protocol: prospective observational cohort study. *BMJ* 2020;**369**:m1985. doi:10.1136/bmj.m1985

3 McGurnaghan SJ, Weir A, Bishop J, *et al.* Risks of and risk factors for COVID-19

For peer review only

disease in people with diabetes: a cohort study of the total population of Scotland. *Lancet Diabetes Endocrinol* Published Online First: 23 December 2020. doi:10.1016/S2213-8587(20)30405-8

4 Barron E, Bakhai C, Kar P, *et al.* Associations of type 1 and type 2 diabetes with COVID-19-related mortality in England: a whole-population study. *Lancet Diabetes Endocrinol* 2020;**8**:813–22. doi:10.1016/S2213-8587(20)30272-2

5 Reilev M, Kristensen KB, Pottegård A, *et al.* Characteristics and predictors of hospitalization and death in the first 11 122 cases with a positive RT-PCR test for SARS-CoV-2 in Denmark: a nationwide cohort. *Int J Epidemiol* 2020;**49**:1468–81. doi:10.1093/ije/dyaa140

6 Petrilli CM, Jones SA, Yang J, *et al.* Factors associated with hospital admission and critical illness among 5279 people with coronavirus disease 2019 in New York City: prospective cohort study. *BMJ* 2020;**369**:m1966. doi:10.1136/bmj.m1966

7 Clift AK, Coupland CAC, Keogh RH, *et al.* Living risk prediction algorithm (QCOVID) for risk of hospital admission and mortality from coronavirus 19 in adults: national derivation and validation cohort study. *BMJ* 2020;**371**:m3731. doi:10.1136/bmj.m3731

8 Hamer M, Gale CR, Kivimäki M, *et al.* Overweight, obesity, and risk of hospitalization for COVID-19: A community-based cohort study of adults in the United Kingdom. *Proc Natl Acad Sci U S A* 2020;**117**:21011–3. doi:10.1073/pnas.2011086117

9 Bergström G, Berglund G, Blomberg A, *et al.* The Swedish CArdioPulmonary BioImage Study: objectives and design. *J Intern Med* 2015;**278**:645–59. doi:10.1111/joim.12384

10 Rolfhamre P, Janson A, Arneborn M, *et al.* SmiNet-2: Description of an internet-based surveillance system for communicable diseases in Sweden. *Eurosurveillance* 2006;**11**:15–6. doi:10.2807/esm.11.05.00626-en

11 Ludvigsson JF. The first eight months of Sweden's COVID-19 strategy and the key actions and actors that were involved. *Acta Paediatr Int J Paediatr* 2020;**109**:2459–71. doi:10.1111/apa.15582

12 Drefahl S, Wallace M, Mussino E, *et al.* A population-based cohort study of socio-demographic risk factors for COVID-19 deaths in Sweden. *Nat Commun* 2020;**11**:5097. doi:10.1038/s41467-020-18926-3

13 Xie J, Zu Y, Alkhatib A, *et al.* Metabolic Syndrome and COVID-19 Mortality Among Adult Black Patients in New Orleans. *Diabetes Care* 2021;**44**:188–93. doi:10.2337/dc20-1714

14 Garg H, Khanna P. Covid and cholesterol (C&C): Something to worry about or much ado about nothing? *Trends Anaesth Crit Care* Published Online First: September 2020. doi:10.1016/j.tacc.2020.09.003

15 Hopkinson NS, Rossi N, El-Sayed_Moustafa J, *et al.* Current smoking and COVID-19 risk: results from a population symptom app in over 2.4 million people. *Thorax* 2021;:thoraxjnl-2020-216422. doi:10.1136/thoraxjnl-2020-216422

For peer review only - http://bmjopen.bmj.com/site/about/guidelines.xhtml

16 Wenzl T. Smoking and COVID-19 - A review of studies suggesting a protective effect of smoking against COVID-19. EU Sci. Hub. doi:10.2760/564217

17 Dillinger JG, Benmessaoud FA, Pezel T, *et al.* Coronary Artery Calcification and Complications in Patients With COVID-19. *JACC Cardiovasc Imaging* 2020;**13**:2468–70. doi:10.1016/j.jcmg.2020.07.004

18 Clark CE, McDonagh STJ, McManus RJ, *et al.* COVID-19 and hypertension: risks and management. A scientific statement on behalf of the British and Irish Hypertension Society. J. Hum. Hypertens. 2021;:1–4. doi:10.1038/s41371-020-00451-x

19 Ludvigsson JF. The first eight months of Sweden's COVID-19 strategy and the key actions and actors that were involved. *Acta Paediatr* 2020;:apa.15582. doi:10.1111/apa.15582

For peer review only

Supplementary Table 1 Definitions and categorization of selected variables.

Variable	Categorization	n (%) missing[a]	n (%) missing in main analyses[b]
**Sociodemographic information**			
Age	Continuous in years	0 (0)	936 (3.1)
Sex	1. Women<br>2. Men	0 (0)	936 (3.1)
Place of birth	1. Not born in Sweden<br>2. Born in Sweden	811 (2.7)	936 (3.1)
Education	1. High school or less<br>2. Vocational education<br>3. University	845 (2.8)	
**Cardiometabolic risk factors**			
Diabetes status	1. Normoglycemia<br>2. Prediabetes (fasting glucose [6.1-6.9 mmol/L or glycated hemoglobin ≥42 mmol/mol and <48 mmol/mol])<br>3. Diabetes diagnosis by physician (self-reported in questionnaire) or glycated hemoglobin ≥48 mmol/mol.	145 (0.5)	1064 (3.6)
Glycated hemoglobin	Continuous in mmol/mol	152 (0.5)	1070 (3.6)
Body mass index	Continuous in kg/m^2	2 (<0.5)	936 (3.1)
Weight status	1. Normal weight<br>2. Overweight<br>3. Obesity	2 (0)	936 (3.1)
Waist-hip ratio	Continuous	1567 (5.2)	2441 (8.1)
Systolic blood pressure	Continuous in mmHg	151 (0.5)	1022 (3.4)
Diastolic blood pressure	Continuous in mmHg	153 (0.5)	1024 (3.4)
Blood pressure level	Level as measured at inclusion in SCAPIS.<br>1. Normotensive (systolic blood pressure <140 mmHg and diastolic blood pressure <90 mmHg)<br>2. Grade 1 hypertension (Systolic blood pressure ≥140 mmHg and <160 mmHg or diastolic blood pressure ≥90 mmHg and <100 mmHg)<br>3. Grade 2 hypertension (systolic blood pressure ≥160 mmHg or diastolic blood pressure ≥100 mmHg).	153 (0.5)	1024 (3.4)
Current smoking	Self-reported in questionnaire.<br>1. No<br>2. Yes	954 (3.2)	1288 (4.3)
Time spent sedentary per day	Self-reported in questionnaire.<br>Continuous (hours per day)	10441 (34.9)	10475 (35)
Coronary artery calcium score by computer tomography	Continuous	1197 (4.0)	1992 (6.6)
Total cholesterol	Continuous in mmol/L	82 (0.3)	1004 (3.4)

Low-density lipoprotein (LDL) cholesterol	Continuous in mmol/L	218 (0.7)	1134 (3.8)
HDL cholesterol	Continuous in mmol/L	85 (0.3)	1005 (3.4)
Creatinine	Continuous in mikromol/L	61 (0.2)	984 (3.3)

a. n (%) missing values out of the total study population (n=29,955)
b. n (%) missing in the main analyses adjusted for age, sex, place of birth and education.

For peer review only

Supplementary Table 2 Characteristics of SCAPIS participants by their status of laboratory-confirmed diagnosis of Covid-19 between January 31 and September 12, 2020. Numbers are shown in n (%) unless otherwise indicated.

	Total study population	Laboratory-confirmed diagnosis of Covid-19
n		
	29955	299
Age, mean (SD)	61.2 (4.5)	60.3 (4.2)
Men	14535 (48.5)	127 (42.5)
Born outside of Sweden	4747 (16.3)	71 (24.1)
Education		
High School or less	2712 (9.3)	26 (8.9)
Vocational	13249 (45.5)	129 (44.0)
University	13149 (45.2)	138 (47.1)
Weight status		
Normal weight	10746 (35.9)	103 (34.4)
Overweight	12850 (42.9)	127 (42.5)
Obesity	6357 (21.2)	69 (23.1)
Body mass index in kg/m^2, mean (SD)	27.0 (4.5)	27.3 (4.7)
Diabetes status		
Normoglycemia	22806 (76.5)	224 (74.9)
Prediabetes	4747 (15.9)	46 (15.4)
Diabetes	2257 (7.6)	29 (9.7)
Waist-hip ratio, mean (SD)	0.9 (0.1)	0.9 (0.1)
Current smoking	3791 (13.1)	23 (8.0)
Sedentary time per day in hours, mean (SD)	6.9 (3.6)	6.6 (3.8)
Blood pressure level		
Normotensive	23335 (78.3)	235 (79.1)
Grade 1 hypertension	5141 (17.3)	48 (16.2)
Grade 2 hypertension	1326 (4.4)	14 (4.7)
Systolic blood pressure, mean (SD)	125.9 (17.0)	126.4 (17.9)
Diastolic blood pressure, mean (SD)	77.5 (10.5)	77.6 (10.7)
Triglycerides in mmol/L, mean (SD)	1.2 (0.8)	1.3 (0.9)
HDL in mmol/L, mean (SD)	1.6 (0.5)	1.6 (0.5)
Total cholesterol in mmol/L, mean (SD)	5.5 (1.1)	5.6 (1)
LDL cholesterol in mmol/L, mean (SD)	3.4 (1.0)	3.5 (0.9)
Glycated hemoglobin in mmol/mol, mean (SD)	36.6 (6.5)	36.8 (6.8)
Creatinine in µmol/L, mean (SD)	77.7 (16.5)	77.3 (22.6)
Coronary artery calcium score, mean (SD)	61.6 (229.5)	39.5 (144.4)

For peer review only

STROBE Statement—Checklist of items that should be included in reports of ***cohort studies***

	Item No	Recommendation	Page
**Title and abstract**	1	(*a*) Indicate the study's design with a commonly used term in the title or the abstract	Title and abstract
		(*b*) Provide in the abstract an informative and balanced summary of what was done and what was found	Abstract
**Introduction**			
Background/rationale	2	Explain the scientific background and rationale for the investigation being reported	p.8
Objectives	3	State specific objectives, including any prespecified hypotheses	p.8
**Methods**			
Study design	4	Present key elements of study design early in the paper	p.8-10
Setting	5	Describe the setting, locations, and relevant dates, including periods of recruitment, exposure, follow-up, and data collection	p.8-10
Participants	6	(*a*) Give the eligibility criteria, and the sources and methods of selection of participants. Describe methods of follow-up	p. 10
		(*b*) For matched studies, give matching criteria and number of exposed and unexposed	
Variables	7	Clearly define all outcomes, exposures, predictors, potential confounders, and effect modifiers. Give diagnostic criteria, if applicable	p. 8-10 Supplementary Material (Supplementary Table 1).
Data sources/ measurement	8*	For each variable of interest, give sources of data and details of methods of assessment (measurement). Describe comparability of assessment methods if there is more than one group	8-10, Supplementary Table 1.
Bias	9	Describe any efforts to address potential sources of bias	p. 8-10
Study size	10	Explain how the study size was arrived at	p.10
Quantitative variables	11	Explain how quantitative variables were handled in the analyses. If applicable, describe which groupings were chosen and why	p.9-10, Supplementary Table 1
Statistical methods	12	(*a*) Describe all statistical methods, including those used to control for confounding	p.10-11
		(*b*) Describe any methods used to examine subgroups and interactions	p.10-11
		(*c*) Explain how missing data were addressed	p.10
		(*d*) If applicable, explain how loss to follow-up was addressed	NA
		(*e*) Describe any sensitivity analyses	NA
**Results**			
Participants	13*	(a) Report numbers of individuals at each stage of study—eg numbers potentially eligible, examined for eligibility, confirmed eligible, included in the study, completing follow-up, and analysed	p.10
		(b) Give reasons for non-participation at each stage	p.10
		(c) Consider use of a flow diagram	NA
Descriptive data	14*	(a) Give characteristics of study participants (eg demographic, clinical, social) and information on exposures and potential	Table 1

For peer review only - http://bmjopen.bmj.com/site/about/guidelines.xhtml

		confounders	
		(b) Indicate number of participants with missing data for each variable of interest	Table 1
		(c) Summarise follow-up time (eg, average and total amount)	p.10
Outcome data	15*	Report numbers of outcome events or summary measures over time	p.11
Main results	16	(*a*) Give unadjusted estimates and, if applicable, confounder-adjusted estimates and their precision (eg, 95% confidence interval). Make clear which confounders were adjusted for and why they were included	p.10-13, Table 1, Table 2
		(*b*) Report category boundaries when continuous variables were categorized	Supplementary Table 1
		(*c*) If relevant, consider translating estimates of relative risk into absolute risk for a meaningful time period	Not deemed relevant
Other analyses	17	Report other analyses done—eg analyses of subgroups and interactions, and sensitivity analyses	p. 10-13
**Discussion**			
Key results	18	Summarise key results with reference to study objectives	p.15
Limitations	19	Discuss limitations of the study, taking into account sources of potential bias or imprecision. Discuss both direction and magnitude of any potential bias	p.16
Interpretation	20	Give a cautious overall interpretation of results considering objectives, limitations, multiplicity of analyses, results from similar studies, and other relevant evidence	p.15
Generalisability	21	Discuss the generalisability (external validity) of the study results	p.15-16
**Other information**			
Funding	22	Give the source of funding and the role of the funders for the present study and, if applicable, for the original study on which the present article is based	p.17

*Give information separately for exposed and unexposed groups.

Note: An Explanation and Elaboration article discusses each checklist item and gives methodological background and published examples of transparent reporting. The STROBE checklist is best used in conjunction with this article (freely available on the Web sites of PLoS Medicine at http://www.plosmedicine.org/, Annals of Internal Medicine at http://www.annals.org/, and Epidemiology at http://www.epidem.com/). Information on the STROBE Initiative is available at http://www.strobe-statement.org.

For peer review only
